# CRISPR/Cas9-Driven Engineering of AcMNPV Using Dual gRNA for Optimized Recombinant Protein Production

**DOI:** 10.3390/v17081041

**Published:** 2025-07-25

**Authors:** Rocco Valente, Joaquín Poodts, Joaquín Manuel Birenbaum, María Sol Rodriguez, Ignacio Smith, Jorge Alejandro Simonin, Franco Uriel Cuccovia Warlet, Aldana Trabucchi, Salvador Herrero, María Victoria Miranda, Mariano Nicolás Belaich, Alexandra Marisa Targovnik

**Affiliations:** 1 Cátedra de Biotecnología, Departamento de Microbiología, Inmunología, Biotecnología y Genética, Facultad de Farmacia y Bioquímica, Universidad de Buenos Aires, Junín 956, Buenos Aires C1113AAD, Argentina; roccovalente838@gmail.com (R.V.); joaquinpoodts@gmail.com (J.P.); joaquinbiren@gmail.com (J.M.B.); solrodriguez.2098@gmail.com (M.S.R.); ignacio.smith@trebebiotech.com (I.S.); 2 Instituto de Nanobiotecnología (NANOBIOTEC), Consejo Nacional de Investigaciones Científicas y Técnicas (CONICET), Universidad de Buenos Aires, Junín 956, Buenos Aires C1113AAD, Argentina; 3 Trebe Biotech SRL, Ruta Nacional 8 KM 225.5, Pergamino, Buenos Aires B2700, Argentina; 4 Laboratorio de Ingeniería Genética y Biología Celular y Molecular, Área Virosis de Insectos, Instituto de Microbiología Básica y Aplicada, Comisión de Investigaciones Científicas de la Provincia de Buenos Aires, Departamento de Ciencia y Tecnología, Universidad Nacional de Quilmes, Roque Sáenz Peña 352, Bernal, Buenos Aires B1876BXD, Argentina; jorge.simonin@unq.edu.ar (J.A.S.); franco.cuccovia@unq.edu.ar (F.U.C.W.); 5 Cátedra de Inmunología, Departamento de Microbiología, Inmunología, Biotecnología y Genética, Facultad de Farmacia y Bioquímica, Universidad de Buenos Aires, Junín 956, Buenos Aires C1113AAD, Argentina; 6 Instituto de Estudios de la Inmunidad Humoral “Prof. Ricardo A. Margni” (IDEHU), Consejo Nacional de Investigaciones Científicas y Técnicas (CONICET), Universidad de Buenos Aires, Junín 956, Buenos Aires C1113AAD, Argentina; 7 Department of Genetics and University, Institute of Biotechnology and Biomedicine (BIOTECMED), Universitat de València, Dr. Moliner 50, Burjassot, 46100 Valencia, Spain; salvador.herrero@uv.es

**Keywords:** CRISPR/Cas9, baculovirus, AcMNPV, genome editing, dual sgRNA, knockout, *Spodoptera frugiperda*, *Rachiplusia nu*, Sf9 cells, expression, optimization

## Abstract

The CRISPR/Cas9 system is a powerful genome-editing tool that is applied in baculovirus engineering. In this study, we present the first report of the AcMNPV genome deletions for bioproduction purposes, using a dual single-guide RNA (sgRNA) CRISPR/Cas9 approach. We used this method to remove nonessential genes for the budded virus and boost recombinant protein yields when applied as BEVS. We show that the co-delivery of two distinct ribonucleoprotein (RNP) complexes, each assembled with a sgRNA and Cas9, into Sf9 insect cells efficiently generated deletions of fragments containing tandem genes in the genome. To evaluate the potential of this method, we assessed the expression of two model proteins, eGFP and HRPc, in insect cells and larvae. The gene deletions had diverse effects on protein expression: some significantly enhanced it while others reduced production. These results indicate that, although the targeted genes are nonessential, their removal can differentially affect recombinant protein yields depending on the host. Notably, HRPC expression increased up to 3.1-fold in *Spodoptera frugiperda* larvae. These findings validate an effective strategy for developing minimized baculovirus genomes and demonstrate that dual-guide CRISPR/Cas9 editing is a rapid and precise tool for baculovirus genome engineering.

## 1. Introduction

The advent of the recombinant nucleic acid technology and its application across various fields of biotechnology have been key to improving human and animal health, agriculture, and the environment. Among all the expression platforms used to produce recombinant proteins, the baculovirus/insect expression system, also known as baculovirus expression vector system (BEVS), stands out for its versatility and flexibility. It allows the efficient production of high-quality proteins at lower costs than mammalian cell systems, for diagnostic, therapeutic, and preventive purposes [1]. Additionally, BEVS offers a high safety profile, as baculoviruses are restricted to arthropods and cannot replicate in vertebrate cells, thereby minimizing biohazard risks. The system also provides a suitable eukaryotic environment for the production of properly folded proteins with complex, native-like post-translational modifications, which are essential for correct structure and biological activity [2,3]. An increasing number of products derived from BEVS have received regulatory approval for use in both human and veterinary medicine [4,5,6].

The BEVS is based on the Autographa californica Nuclear Polyhedrosis Virus (AcMNPV), a prototypical species (*Alphabaculovirus aucalifornicae*) belonging to the *Baculoviridae* family. It infects 32 species of Lepidoptera and can infect commercially available insect cell lines derived from *Spodoptera frugiperda* (Sf21 and the subclone Sf9) and *Trichoplusia ni* (Tn-5) [7]. The AcMNPV genome consists of a circular double-stranded DNA molecule of 134 kbp encoding around 155 predicted protein genes and is fully sequenced [8,9,10]. The functions of several of its genes are still poorly understood, but they are generally believed to play a role in the virus’s ability to propagate [11,12]. In nature, baculoviruses mainly infect the larvae of their hosts when they feed on plants contaminated with the virus [13]. During their biphasic viral cycle, some baculoviruses (including AcMNPV) produce two distinct phenotypes: occlusion-derived viruses (ODVs) and budded viruses (BVs). ODVs are involved in horizontal transmission between insects through protein structures known as occlusion bodies (OBs), where the virus is embedded. In contrast, BVs are responsible for spreading the infection from cell to cell [14]. For biotechnological applications, it is important to note that BVs are the virions required for infecting cell cultures in vitro. BVs can also be used to infect larvae via intrahemocoelic injections, making OBs unnecessary for BEVS.

The use of Lepidopteran larvae as “biofactories” has been explored as an alternative to cell cultivation. This host yields higher yields than insect cells at a significantly lower cost, due to the larvae’s minimal commercial value and low maintenance and breeding requirements [15,16]. Nowadays, several products for veterinary use manufactured using insect larvae are already available on the market. Despite the significant advantages of using larvae, insect cell cultivation remains the primarily accepted expression system to produce recombinant proteins for therapeutic purposes in human health [1]. For all these reasons, the development and optimization of the platform should focus on protein production both in cultures and in Lepidoptera larvae.

Considerable progress has been made in understanding baculovirus biology in recent decades. It has been demonstrated that 45% of the AcMNPV genome may be nonessential for BV production [17,18]. Specifically, genes coding for *per os* infectivity factors (PIFs) and others associated with ODV envelope formation, as well as genes involved in OB formation, are only required for oral infection of insects, not for cell culture infection or the intrahemocoelic injection of Lepidoptera larvae [14,19]. The deletion of nonessential genes has been shown to improve the expression of recombinant proteins [11]. There is significant potential to optimize this platform by obtaining minimized genomes, as these genes could hinder the production of recombinant proteins or compete for cellular resources [19,20].

Since the system’s development, AcMNPV has been genetically modified using various strategies to obtain more stable genomes and higher expression yields [11]. In this sense, the deletion of genes coding for viral chitinase and cathepsin—both responsible for larval liquefaction—along with the deletion of nonessential genes associated with ODVs such as p26, p10, and p74, has improved the quality and increased the expression levels of heterologous proteins produced in insect cells infected with recombinant baculoviruses [20,21]. Despite being a widely used method, the traditional techniques for genome manipulation based on homologous recombination are time-consuming and require several rounds of selection, which are imprecise and challenging, especially when large regions need to be removed [22]. In particular, the λ Red recombination system is currently widely used to optimize the AcMNPV genome by eliminating nonessential genes for BV systemic infection or genes with unknown functions [11,12,18,23,24,25,26].

With the introduction of the CRISPR/Cas9 genome editing system in 2013, new opportunities have emerged in the field of biotechnology. This technology allows for easy and rapid editing of large viral genomes (mutations, insertions, deletions, and DNA additions) [22]. The primary system consists of the Cas9 endonuclease from *Streptococcus pyogenes*, which is capable of catalyzing the hydrolysis of double-stranded DNA, as well as a single-guide RNA (sgRNA) that directs the Cas9 enzyme to a specific sequence for cleavage. The resulting damage to the double-stranded DNA molecule (DSB, for DNA double-strand break) is recognized and repaired by the cellular machinery [9]. Recently, this technology has been applied for the first time to edit genes within the AcMNPV genome [9,27]. Unlike λ red recombinant systems, this strategy allows direct modification of the viral genome without requiring passage through bacteria. Additionally, it eliminates the use of antibiotics for mutant selection [28]. Varying levels of success have been achieved depending on the gene, through the transfection of the Cas9-sgRNA complex in the presence of a single sgRNA. The resulting mutations consist of small insertions or deletions, leading to frameshift mutations and premature stop codons. These genetic disruptions are detectable only through sequencing and lead to pseudogenization—the process by which a gene loses its protein-coding ability due to mutations [9]. Although this single-guide RNA methodology is well-established, the application of two customized sgRNAs to simultaneously remove multiple tandem genes, including regulatory regions and promoters, remains unexplored. This would prevent transcriptional complex formation and result in substantial energy savings for the cells. In addition, this strategy would allow the mutant virus to be detected with just a polymerase chain reaction assay (PCR). In this framework, we developed and validated a highly efficient CRISPR/Cas9 editing process to separately remove four genomic baculovirus regions containing tandem nonessential genes involved in BV production. Therefore, after confirming that replication was not impaired, we used all mutants to evaluate recombinant protein expression in both insect cells and, for the first time, in larvae. We selected two model proteins for evaluation: enhanced green fluorescent protein (eGFP), a well-characterized fluorescent reporter enabling rapid quantification of expression levels; and horseradish peroxidase isoenzyme C (HRPc) fused to the GP64 signal peptide, used as a proxy for complex post-translational modification and export machinery. These proteins serve as robust benchmarks: eGFP for soluble intracellular production and HRPc for secreted functionality. This approach enables systematic assessment of baculovirus-driven expression under genomic modifications and provides a valuable tool for improving the baculovirus genome and enhancing protein production.

## 2. Materials and Methods

### 2.1. Cell Culture and Insects

The Sf9 insect cell line was cultured in Sf900 II (Thermo Fisher Scientific, Waltham, MA, USA) supplemented with 1% (*v*/*v*) fetal bovine serum (FBS) and 1% (*v*/*v*) antibiotic-antimycotic solution (Thermo Fisher Scientific) at 27 °C. Larvae of *S. frugiperda* and *Rachiplusia nu* were provided by Agidea (Pergamino, Argentina). The larvae were reared in a chamber at 23–25 °C with 70% humidity and fed a specially formulated artificial diet.

### 2.2. Parental Bacmid Bac-eGFP/HRPC and Parental Virus Ac-eGFP/HRPC Construction

The synthetic HRPc generated by British Biotechnologies Ltd. was kindly provided by Dr. P.E. Ortiz de Montellano. This coding sequence was previously cloned into the pAcGP67 vector under the baculoviral glycoprotein 64 leader peptide (GP64, also known as GP67) [29]. Using pAcGP67-HRPc as a template, the HRPc fused to the GP64 signal peptide was amplified by PCR using two specific primers (Fw-HRPcEcoR1 and Rv-HRPcEcoR1) (see Table S1 for primer sequences), which introduced the EcoRI site. The PCR conditions (50 μL final volume) were as follows: 200 nM of each primer, 1 × PFU buffer, 0.3 mM of each dNTP, and 2.5 U PFU polymerase (Promega, Madison, USA). The PCR program was 95 °C for 6 min, 95 °C for 30 s, 56 °C for 90 s, and 72 °C for 1 min/kb × 30 cycles. An additional extension step of 72 °C for 5 min was then applied. After the reaction, the PCR product was purified using the PCR Wizard^TM^ SV gel and PCR Clean-up System (Promega, Madison, WI, USA). The HRPc sequence fused to the GP64 signal peptide was then cloned using EcoRI sites into pFBD-p10eGFP-polh-pSeL-x under polh-pSeL chimera promoter to construct pFBD-eGFP/HRPC. The plasmid pFBDp10eGFP-polh-pSeL-x also carried the eGFP under p10 promoter [30].

The recombinant bacmid Bac-eGFP/HRPc (parental bacmid for genome editing) was obtained by using the Bac-to-Bac^®^ baculovirus expression system (Thermo Fisher Scientific), following the manufacturer’s instructions. The pFBD-eGFP/HRPc vector was used to transform the chemically competent E. coli DH10Bac™ strain (Thermo Fisher Scientific) by heat shock to generate the recombinant bacmid by transposition. The bacmid DNA was isolated following the manufacturer’s instructions and used to obtain the recombinant baculovirus *Ac-eGFP/HRPc* (parental virus). For this purpose, 1 × 10^6^ Sf9 cells were transfected [31] with the bacmid by using Cellfectin II Reagent (Thermo Fisher Scientific). After a 6-day incubation at 27 °C, the Ac-eGFP/HRPc virus was harvested from the cell culture supernatant and centrifuged at 500× *g* for 10 min. Transfection efficiency was determined by measuring eGFP expression by fluorescence under UV light. After three amplification steps, the virus titer was determined using an end-point dilution assay. The infectious dose 50 (DI_50_) was converted to plaque-forming units per milliliter (pfu/mL) using a coefficient of 0.69 [32]. The amplified virus stock was used to produce the eGFP and HRPc in further experiments.

### 2.3. Knockout in Bac-eGFP/HRPc

A CRISPR-Cas-based approach was implemented using a pair of sgRNAs to induce targeted gene knockouts within the baculovirus genome. Deletion or, alternatively, gene disruption was carried out on promoter regions or open reading frames (ORF) belonging to genes whose alteration is described as not compromising BV formation and infectivity [14]. Promoters were predicted by bioinformatics analysis (https://www.fruitfly.org/seq_tools/promoter.html; last accessed on 15 June 2025). Given the aforementioned criteria and considering the transcriptomic data [33], four groups of ORFs were selected for editing in independent assays: *Ac15(egt)-Ac16(*ODV-E26*)*, *Ac129(*p24*)-Ac130-Ac131*(pp34), *Ac136(*p26*)-Ac137*(p10)-*Ac138*(*p74*) and *Ac148* (ODV-E56)-*Ac149*-*Ac150*. Given the nature of the AcMNPV genome (GenBank accession number: L22858.1) and the overlap of functional gene elements, deletion must be performed when it does not affect the expression of an essential gene. Editing was carried out on the “parental bacmid” Bac-eGFP/HRPc, which can replicate in Escherichia coli, after purification, and give rise to AcMNPV virions in Sf9 cells, as described above.

To edit each group of genes, two sgRNAs were designed using the CHOPCHOP bioinformatics program through its online platform (https://chopchop.cbu.uib.no/ last accessed on 15 June 2025) (Table 1). The sgRNAs were selected based on efficiency, prioritizing those with high predicted on-target activity, minimal off-target effects, approximately 50% GC content, and the potential to remove the maximum possible portion of the ORF without harming neighboring essential genes. All were synthesized using the Engen^®^ sgRNA Synthesis Kit, S. pyogenes (New England Biolabs, Ipswich, MA, USA), following the manufacturer’s protocols. The sgRNA was then synthesized, purified using RNA Cleanup Kit (50 μg) (New England Biolabs), and quantified using Qubit equipment (Thermo Fisher Scientific).

Subsequently, the RNP complex (ribonucleoprotein, sgRNA-Cas9) was formed by incubating 100 ng of each sgRNA and 1 µg of the EnGen Spy Cas9 NLS Cas9 Nuclease (New England Biolabs) for 5 min at 37 °C. The resulting complex was then used for cell transfection assays.

The efficacy of the RNP was analyzed individually for each guide with its corresponding DNA fragment using in vitro Cas9 digestion. The fragments were obtained by PCR, as mentioned above, using specific oligonucleotides (Table S1). After PCR fragment purification, DNA (200 ng) was incubated with the corresponding RNP complex in 15 µL final volume in the presence of NEBuffer 3.1 (New England Biolabs, Ipswich, MA, USA) and 7.5 µg of BSA (Promega). The cutting efficiency of the enzyme at the desired site was visualized through agarose gel electrophoresis (Figures S1–S3).

For edition in insect cells, 1 µg of the parental bacmid “Bac-eGFP/HRPc” and each pair of RNP were mixed with Cellfectin^®^ II Reagent (Thermo Fisher Scientific) in 100 µL of Sf900 II medium without FBS. The mixture was incubated in three separate tubes in the dark for 20 min at room temperature. Then, the three solutions were mixed and incubated for an additional 20 min. The solution was finally used to transfect 1 × 10^6^ Sf9 cells. After incubating for 5 h at 27 °C, the medium was replaced by Sf900 II medium supplemented with 1% FBS and incubated for 6 days at 27 °C. Transfection efficiency was confirmed by microscopic observation of eGFP and HRPc activity in the supernatant culture. The supernatant containing the BVs was clarified by centrifugation at 500× *g* and then stored at 4 °C. This procedure was repeated for each gene group in independent assays.

**Figure 1:**
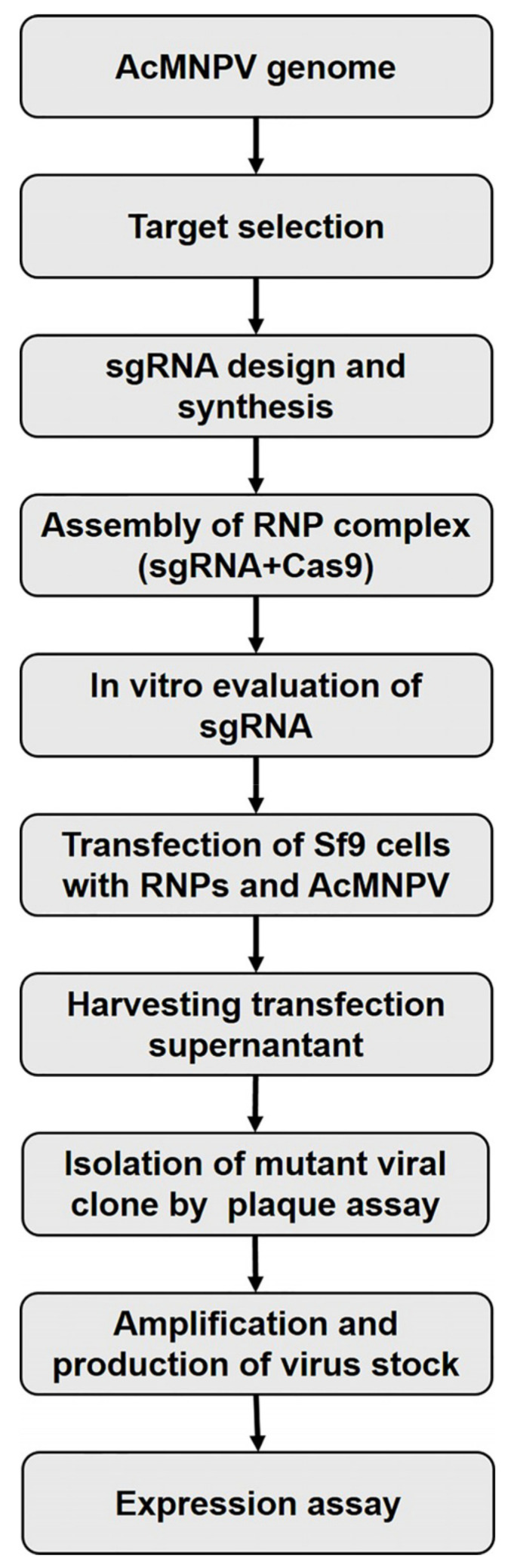
Schematic workflow of CRISPR/Cas9-mediated genome editing using dual sgRNAs in baculovirus. After target selection, two sgRNAs were synthesized and Sf9 cells were co-transfected with the AcMNPV bacmid. Mutant clones were isolated and used to assess the expression of eGFP and HRPc.

Individual viruses derived from each transfection event were isolated from the supernatants (containing a mixture of edited and nonedited viruses) using the plaque assay [34]. Each clone was identified under microscopic observation of eGFP. Ten individual clones were amplified in Sf9 cells, and a fraction (10 μL) was treated with PrepMan^®^ Ultra reagent (Applied Biosystems™, Foster City, CA, USA) to be used for PCR amplification of the targeted region using specific primers flanking the knockout location (Table S1). PCR products were analyzed by agarose gel electrophoresis and one representative clone was purified and sequenced using the Sanger method (“Ricardo Gutierrez” Hospital, Buenos Aires, Argentina) to confirm the presence of mutations in the protospacer adjacent motif (PAM)-flaking region. The parental (Ac-eGFP/HRPc) and the edited (*Ac-eGFP/HRPc^∆Ac15-Ac16^*, *Ac-eGFP/HRPc^∆129-Ac131^*, *Ac-eGFP/HRPc^∆136-Ac138^*, *Ac-eGFP/HRPc^∆Ac148-Ac150^*) baculoviruses were further amplified to high-titer stocks by infecting Sf9 cells at multiplicity of infection (MOI) of 0.02. Figure 1 shows an overview of the methodology used.

### 2.4. Effect of Genome Editing on Viral Replication

The effect of genome editing on viral replication was evaluated by quantitative real-time PCR (qPCR) using two specific primers (Fw-ie1; Rv-ie1) (Table S1) and the Master Mix qPCR Sybr/ROX (Productos Biológicos, Buenos Aires, Argentina) according to the manufacturer’s instructions, on a Step one Real-Time PCR System (Applied Biosystems™). For this purpose, supernatant expression at 4 days post infection (dpi) was treated with Prepman^TM^ reagent (Applied Biosystems) following the manufacturer’s instructions and quantified by comparing the obtained Ct values against a standard curve of ie1 viral gene (1 × 10^2^ copies/µL^−1^ × 10^8^ copies/µL) previously cloned into the pGemT-Easy vector (Promega) [35]. The viral titers were expressed as genome copies of baculoviruses per milliliter (copies/mL). Nonedited baculovirus clones were also included as a control. All experiments were performed in triplicate and analyzed using one-way ANOVA (*p* < 0.05).

### 2.5. Sf9 Cell Culture and Infection

The effect of genome editing on recombinant protein expression was evaluated in SF9 cells. Expression assays were assessed at MOIs 0.5 and 5 pfu per cell on 5 × 10^5^ Sf9 cells in 0.5 mL of medium using 12-well dishes. A negative control plate containing uninfected cells was included in the analysis. The cells were infected with the different recombinant baculoviruses (parental or edited). The plates were incubated in the dark at 27 °C for 3 or 4 days for eGFP and HRPc expression, respectively. The cell culture was collected by low-speed centrifugation (500× *g* for 5 min). Then, the pellet and the supernatant were separated and kept at −20 °C until quantification of eGFP at 3 dpi (from cell pellet) and HRPc at 4 dpi (from cell supernatant). To evaluate eGFP expression, the cell pellet was resuspended in lysis buffer (50 mM Tris-HCl pH 7.5; 100 mM NaCl; 1 mM DTT; 5% glycerol). The samples were centrifuged at 12,000× *g*, and the supernatant was used to determine eGFP expression.

### 2.6. Larvae Infection

The effect of genome editing on recombinant protein expression was evaluated in *S. frugiperda* and *R. nu* larvae. Fourth instar *S. frugiperda* and *R. nu* larvae were infected with either edited or parental recombinant baculovirus in individual experiments via the intrahemocoelic route. For inoculation, the larvae were anesthetized by immersion in an ice-water bath for 10 min. A total of 5 × 10^5^ pfu was injected in a final volume of 50 µL. An hour after injection, the larvae were transferred to the rearing room at 27 °C and fed. At 3 dpi, the larvae were harvested and homogenized in groups of five larvae (*n* = 10) in the presence of 2.5 mL extraction buffer (50 mM sodium phosphate buffer, pH 6.0, 5 mM EDTA, 0.2 mg ml_1 PMSF, 150 mM KCl with glutathione crystals) per gram of larvae to obtain larval extracts. The homogenates were centrifuged at 12,000× *g* at 4 °C, and the supernatant was collected to evaluate HRPc and eGFP expression levels. Uninfected larvae were included in the analysis as a negative control.

### 2.7. Analysis of eGFP Expression in sf9 Pellet and Larval Extract

The eGFP was measured by determining the emission of fluorescence in a microplate reader (λ excitation: 485 nm and λ emission: 535 nm; TECAN Infinite M200Pro, Tecan Group Ltd., Männedorf, Switzerland). Values were expressed as the relative eGFP fluorescence intensity (fluorescent units per ml, FU/mL), with a value of 1 corresponding to the maximum intensity obtained with the control. For all the experiments, the reported values corresponded to at least three independent replicates. Statistical analyses were performed using Dunnett’s multiple comparison test in the GraphPad Prism program (version 8.0.1, GraphPad Software Inc., San Diego, CA, USA). Fluorescence in cells and larvae was monitored by fluorescence microscopy using an inverted fluorescence microscope (Carl Zeiss, Jena, Germany) and Leica MZ10 F stereomicroscope (Leica Microsystems GmBH, Wetzlar, Germany), respectively.

### 2.8. Analysis of HRPc Activity in sf9 Cell Expression Supernatant and Larval Extract

HRPc activity was measured by assessing guaiacol oxidation in a reaction mixture containing 30 mM guaiacol and 25 mM hydrogen peroxide in 100 mM potassium phosphate buffer (pH 7.0). Oxidation was initiated by adding a 10 mL sample to the 1 mL reaction mixture. Absorbance was measured at 470 nm within 1.5 min, and activity was calculated as described by [36]. For all the experiments, the reported values correspond to at least three independent replicates. Values were expressed as the relative HRPc activity (U/mL), with a value of 1 corresponding to the maximum intensity obtained with the control. For all the experiments, the reported values corresponded to at least three independent replicates. Statistical analyses were performed using Dunnett’s multiple comparison test in the GraphPad Prism program (version 8.0.1, GraphPad Software Inc., San Diego, CA, USA).

## 3. Results

### 3.1. CRISPR-Mediated Knockout in Bac-eGFP/HRPc Using Dual sgRNA

**Figure 2:**
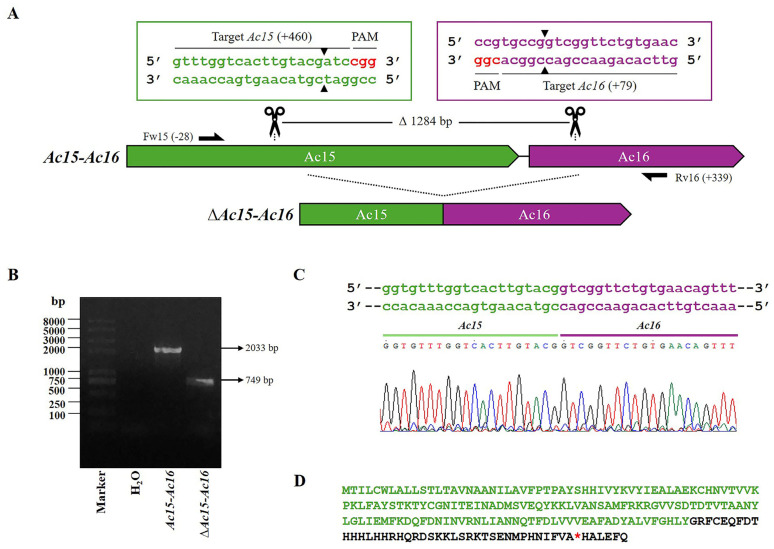
CRISPR dual-sgRNA knockout of *Ac15*-*Ac16* in Bac-eGFP/HRPc. (**A**) Schematic representation of the *Ac15*-*Ac16* genomic locus before and after CRISPR editing. The *Ac15* and *Ac16* are represented in green and purple, respectively. After deletion of the *Ac15*-Ac*16* fragment, the resulting pseudogene consists of the 5′ portion of *Ac15* (green) and the 3′ portion of *Ac16* (purple). Gene-specific primers used for PCR are indicated by black arrows. DNA excision is marked with scissors and a triangle (▲). The hybridization positions of primers and sgRNA targets are shown in parentheses (+/−), using the ATG start codon of each ORF as the reference point. PAM sequences (red nucleotides) are highlighted. (**B**) Identification of edited clones by PCR using specific primers (Fw-*Ac15* and Rv-*Ac16*). The lane labeled “*Ac-eGFP/HRPc*” corresponds to the PCR product amplified from the unedited parental virus (2033 bp). The lane labeled “*Ac-eGFP*/*HRPc^ΔAc15-Ac16^*” corresponds to the PCR product amplified from the edited virus (749 bp). Marker: Trans 2K Plus (TransGen Biotech, Beijin, China). (**C**) Sanger sequencing chromatogram of the edited virus. Only the flanking regions of the knockout are shown. (**D**) Amino acid sequence of the truncated aberrant protein originating from the fusion of *Ac15* and *Ac16*. The conserved N-terminal region from *Ac15* is shown in green, followed by a frameshift-derived sequence (i.e., a sequence resulting from a mutation that alters the original reading frame) leading to a premature stop codon (*). Not at scale.

**Figure 3:**
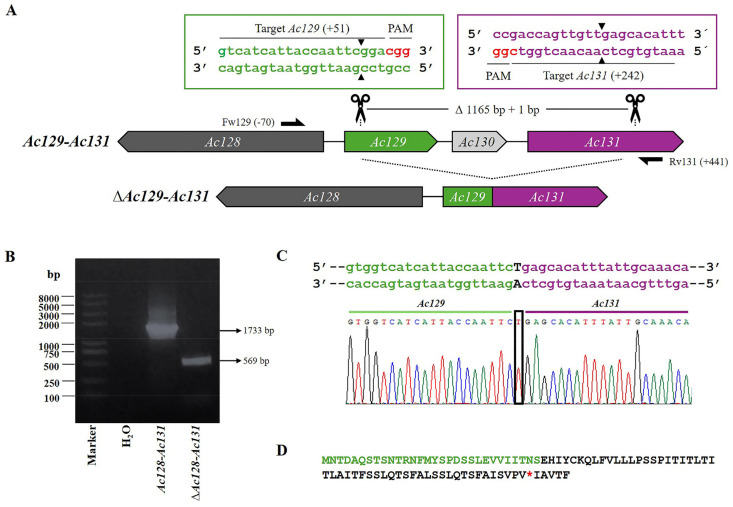
CRISPR dual-sgRNA knockout of *Ac129*-*Ac131* in Bac-eGFP/HRPc. (**A**) Schematic representation of *Ac129*-*Ac131* genomic locus before and after CRISPR editing. The non-edited *Ac128* is shown as a dark grey arrow, while *Ac129*, *Ac130*, and *Ac131* are represented in green, light gray, and purple, respectively. After deletion of the *Ac129-Ac131* fragment, the resulting pseudogene consists of the 5′ portion of *Ac129* (green) and the 3′ portion of *Ac131* (purple). *Ac130* (light grey) is completely removed. Gene-specific primers used for PCR are indicated by black arrows. DNA excision is marked with scissors and a triangle (▲). The hybridization positions of primers and sgRNA targets are shown in parentheses (+/−), using the ATG start codon of each ORF as the reference point. PAM sequences (red nucleotides) are highlighted. (**B**) Identification of edited clones by PCR using specific primers (Fw-*Ac129* and Rv-*Ac131*). The lane labeled “*Ac-eGFP/HRPc*” corresponds to the PCR product amplified from the unedited parental virus (1733 bp). The lane labeled “*Ac-eGFP/HRPc^ΔAc129-Ac131^*” corresponds to the PCR product amplified from the edited virus (569 bp). Marker: Trans 2K Plus (TransGen Biotech). (**C**) Sanger sequencing chromatogram of the edited virus. Only the flanking regions of the knockout are shown. A single nucleotide insertion (T) at the fusion is indicated with a square. (**D**) Amino acid sequence of the truncated aberrant protein originating from the junction of *Ac129* and *Ac131*. The conserved N-terminal region from *Ac129* is shown in green, followed by a frameshift-derived sequence that leads to a premature stop codon (*). Not at scale.

**Figure 4:**
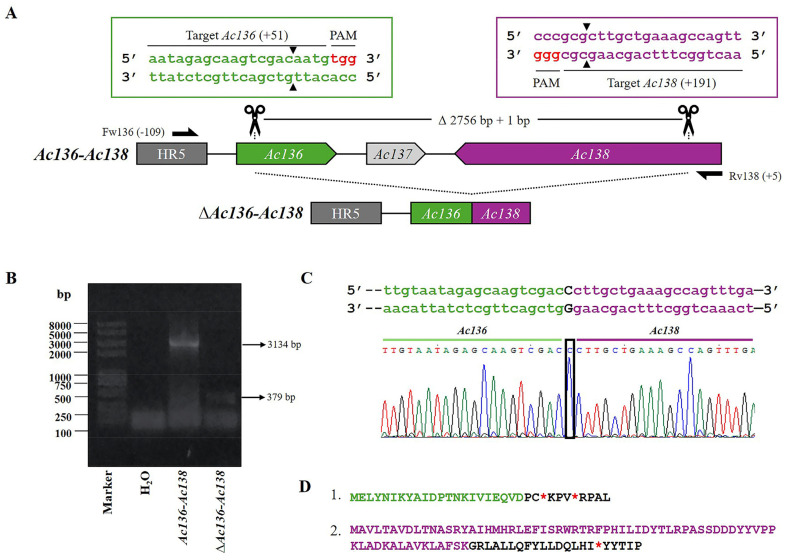
CRISPR dual-sgRNA knockout of *Ac136*-*Ac138* in Bac-eGFP/HRPc. (**A**) Schematic representation of the *Ac136-Ac138* genomic locus before and after CRISPR editing. The non-edited HR5 is shown as a dark grey arrow, while *Ac136*, *Ac137*, and *Ac138* are represented in green, light gray, and purple, respectively. After deletion of the *Ac136*-Ac*138* fragment, the resulting pseudogene consists of the 5′ portion of *Ac136* (green) and the 3′ portion of *Ac138* (purple). *Ac137* (light grey) is completely removed. Gene-specific primers used for PCR are shown as black arrows. DNA excision is marked with scissors and a triangle (▲). The hybridization positions of primers and sgRNA targets are shown in parentheses (+/−), using the ATG start codon of each ORF as the reference point. PAM sequences (red nucleotides) are highlighted. (**B**) Identification of edited clones by PCR using specific primers (Fw-*Ac136* and Rv-*Ac138*). The lane labeled “*Ac-eGFP/HRPc*” corresponds to the PCR product amplified from the unedited parental virus (3134 bp). The lane labeled “*Ac-eGFP/HRPc^ΔAc129-Ac131^*” corresponds to the PCR product amplified from the edited virus (379 bp). Marker: Trans 2K Plus (TransGen Biotech). (**C**) Sanger sequencing chromatogram of the edited virus. Only the flanking regions of the knockout are shown. A single nucleotide insertion (c) at the junction is indicated with a square. (**D**) Amino acid sequence of the truncated aberrant protein originating from the fusion of *Ac13*6 and *Ac138*. 1. The conserved N-terminal region from *Ac129* is shown in green, followed by a frameshift-derived sequence that leads to a premature stop codon (*). 2. The conserved N-terminal region from *Ac138* is shown in green, followed by a frameshift-derived sequence that leads to a premature stop codon (*). Not at scale.

**Figure 5:**
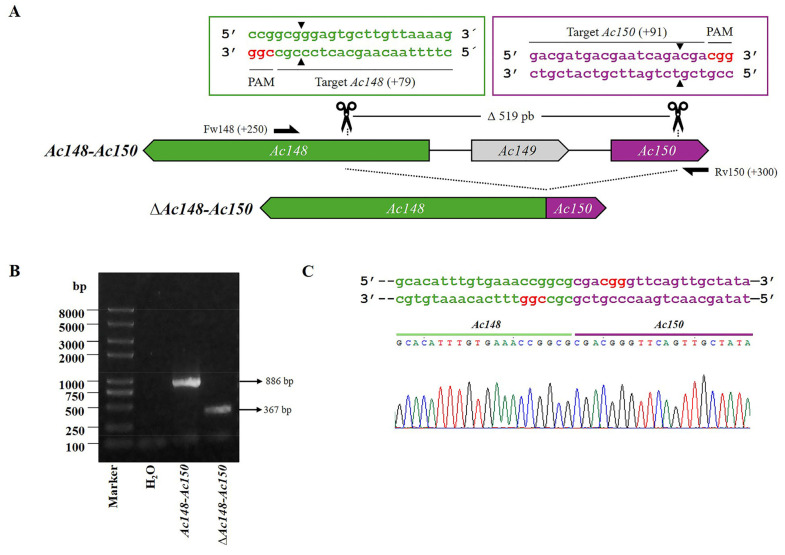
CRISPR dual-sgRNA knockout of *Ac148*-Ac*150* in Bac-eGFP/HRPc. (**A**) Schematic representation of *Ac148*-Ac*150* genomic locus before and after CRISPR editing. The edited *Ac148*, *Ac149*, and *Ac150* are represented in green, light gray and purple, respectively. After deletion of the *Ac148-Ac150* fragment, the resulting pseudogene is composed of the 5′ portion of *Ac148* (green) and the 3′ portion of *Ac150* (purple). *Ac149* (light grey) is completely removed. Gene-specific primers used for PCR are indicated by black arrows. DNA excision is marked with scissors and a triangle (▲). The hybridization positions of primers and sgRNA targets are shown in parentheses, using the ATG start codon of each ORF as the reference point. PAM sequences (red nucleotides) are highlighted. (**B**) Identification of edited clones by PCR using specific primers (Fw-*Ac148* and Rv-*Ac150*). The lane labeled “*Ac-eGFP/HRPc*” corresponds to the PCR product amplified from the unedited parental virus (886 bp). The lane labeled “*Ac-eGFP/HRPc^ΔAc12-Ac131^*” corresponds to the PCR product amplified from the edited mutant virus (367 bp). Marker: Trans 2K Plus (TransGen Biotech). (**C**) Sanger sequencing chromatogram of the edited virus. Only the flanking regions of the knockout are shown. Not at scale.

The recombinant bacmid, Bac-eGFP/HRPc, which served as template for generating gene-defective virions, was constructed using the Bac-to-Bac system. It was subsequently employed to construct the virus, *Ac-eGFP/HRPc*, which encodes two proteins: eGFP (intracytoplasmic) and HRPc (exported). To evaluate the possibility of removing the largest proportion of potentially dispensable genes from the bacmid using two sgRNA and Cas9, four regions were selected: *Ac15-Ac16; Ac129-Ac130-Ac131* (region named *Ac129-Ac131*); *Ac136-Ac137-Ac138* (region named *Ac136-Ac138*); and *Ac148-Ac149-Ac150* (region named *Ac148-Ac150*). The selection criteria were based on previous transcriptomic data [33]. In this regard, regions showing high transcriptional activity, which do not affect BV production, were selected for editing. To this end, two specific sequence targets were selected and their respective sgRNAs were designed to be located on opposite strands [37] (Table 1). According to the bioinformatics analysis performed using the CHOPCHOP online tool, the sgRNAs did not show any off-target effects. Figure 2, Figure 3, Figure 4 and Figure 5 show a schematic representation of each group of edited genes and the location of the sgRNAs. Once generated, the sgRNAs were complexed with Cas9 to form two RNPs and validated through in vitro testing, involving digestion of PCR fragments followed by electrophoretic analysis (Figures S1–S3). All sgRNAs tested were able to cleave the target fragment with varying degrees of efficiency in the in vitro condition tested, as undigested bands were observed along with digestion products on the gel. Only sgRNA-*Ac15*, sgRNA-*Ac136*, and sgRNA-*Ac138* achieved complete fragment digestion. Genome editing was then performed through the direct transfection of both sgRNA together with Bac-eGFP/HRPc. After 6 days, the supernatant was harvested, and the mutant clone viruses were isolated via a plaque assay, multiplied in Sf9 cells, and genotyped by PCR using specific primers (Table S1) that flank the region to be deleted. The editing efficiency, calculated as the ratio of mutant clones isolated to parental clones, varied across the different selected targets: 10% for the *Ac15-Ac16* and *Ac129-Ac131*; 30% for *Ac136-Ac138*; and 20% for *Ac148-Ac150*. To confirm the deletion, one representative clone of each edited regions was Sanger sequenced, and the chromatograms are shown in Figure 2, Figure 3, Figure 4 and Figure 5. All edited fragments exhibited the expected deletion upstream of the PAM site.

First, the region *Ac15-Ac16* was edited (Figure 2A), generating the *Ac-eGFP/HRc^∆Ac15-Ac16^* virus. PCR amplification of the affected locus resulted in a 749 bp fragment, as opposed to the 2033 bp amplicon observed in the nonedited locus (Figure 2B). The eliminated segment of 1284 bp includes the theoretical *Ac16* promoter, which is located in the intergenic region between the *Ac15* and *Ac16* ORFs, as predicted by bioinformatics analysis. As a result of the editing, the *Ac15* ORF generated a truncated protein, with a premature stop codon located within its fusion with the *Ac16* ORF (Figure 2C,D). The theoretical promoter region of *Ac15* could not be removed, as it overlaps with the 3′ end of the preceding essential gene, *Ac14*, which encodes the Lef-1 protein. Additionally, the 5′ end of *Ac15* cannot be removed as it contains the theoretical promoter of *Ac14*. Meanwhile, the protein derived from *Ac16* is expected to be aborted, as its promoter region has been removed. Table S2 shows the predicted promoters and their locations and scores.

Then, in an independent experiment, the region *Ac129-Ac131* was edited (Figure 3A) to generate *Ac-eGFP/HRPc^∆Ac129-Ac131^*, and the PCR analysis of the mutated clones resulted in a fragment of 569 bp, in contrast to the expected 1733 bp in the nonedited locus (Figure 3B). As a result of the modification, 1165 bp were eliminated, leading to the partial deletion of the *Ac129* ORF and the complete removal of the *Ac130* ORF and their promoter predicted region. The loss of *Ac129* resulted in the production of a truncated protein, with a premature stop codon located within its fusion with *Ac131*. Additionally, the elimination of the *Ac130* ORF further altered gene organization, likely preventing *Ac131* protein expression by removing its theoretical promoter, which bioinformatics analysis suggests overlapped with the 3′ end of *Ac130*. The promoter region of *Ac129* could not be removed, as it overlaps with the 5′ end of the essential gene *Ac128* that encodes the GP64 protein, a key factor in BV propagation. Furthermore, a + 1bp insertion occurred at the junction site between the two fragments, resulting from the action of the cell repair machinery following the cut made by Cas9 (Figure 3C). As a result of the editing, the fusion of the N-terminus of *Ac129* with the C-terminus of *Ac131* generated a predicted protein in which the *Ac129* portion remained in-frame, but a frameshift occurred at the junction with *Ac131*, leading to a premature stop codon (Figure 3D).

Moreover, the *Ac136-Ac138* tandem ORFs were edited to generate *Ac-eGFP/HRPc^∆Ac136-Ac138^* (Figure 4A). The PCR from the mutated clones produced a fragment of 379 bp, whereas amplification from *Ac-eGFP/HRPc* resulted in a 3134 bp fragment (Figure 4B). The deleted 2756 bp included a 3′ fragment of *Ac136*, the complete *Ac137* ORF, and its theoretical promoter, which overlapped with *Ac136*, as well as a 3′ fragment of *Ac138*. An insertion of 1 bp also occurred as a result of the repair process (Figure 4C). In addition, the predicted *Ac136 and Ac138* promoters were not removed as they overlapped with the homologous region 5 (HR5) and the essential gene *Ac139*, which encodes ME53, respectively. As a result of the editing, the fusion of the N-terminus of *Ac136* and *Ac138* generated two possible proteins in opposite orientations. On the one hand, the *Ac136* portion remained in-frame, but a frameshift occurred at the junction with *Ac138*, leading to a premature stop codon. On the other hand, the *Ac138* portion remained in-frame, but a frameshift again occurred at the junction, also resulting in a premature stop codon (Figure 4D).

In addition, the region named *Ac148*-*Ac150* was edited to generate *Ac-eGFP/HRPc^∆Ac148-Ac150^* (Figure 5A). The PCR from mutated clones resulted in a 367 bp fragment, whereas amplification from *Ac-eGFP/HRPc* produced a segment of 886 bp (Figure 5B). Since the *Ac149* gene was completely removed, the theoretical promoters of *Ac148* and *Ac150* genes were also eliminated due to their overlap with the 3′and 5′ ends of *Ac149*, respectively. The promoter of *Ac149* was not identified because the bioinformatics predictor program failed to detect its location, likely due to its non-canonical sequence. However, it is probably located in the intergenic region with *Ac150* or overlapping with the 3′end of *Ac150*. Therefore, the editing probably removed the *Ac149* promoter. As a result of the editing, none of the three proteins were expected to be expressed (Figure 5C).

### 3.2. Impact of Gene Edition by Cas9 Using Dual sgRNA on Baculovirus Infectivity and Replication in Cultured sf9 Cells

**Figure 6:**
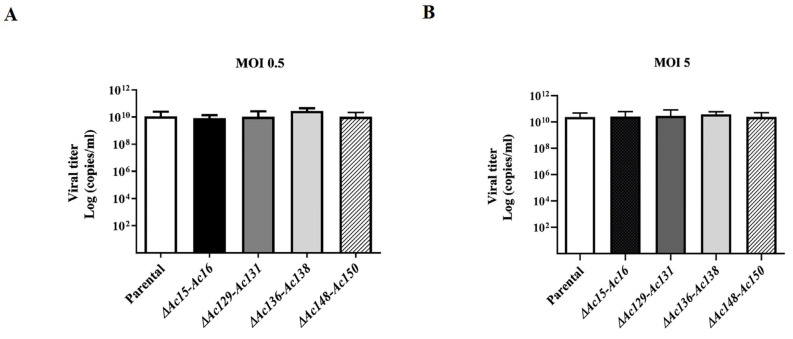
Effect of genome editing on AcMNPV replication. Supernatants were collected from Sf9 cell cultures, derived from cells infected with mutants of AcMNPV (*Ac-eGFP/HRPc^∆Ac15-Ac16^*, *Ac-eGFP/HRPc^∆Ac129-Ac131^*, *Ac-eGFP/HRPc^∆Ac136-Ac138^*, and *Ac-eGFP/HRPc^∆Ac148-Ac150^*, mentioned as *∆Ac15-Ac16*, *∆Ac129-Ac131*, *∆Ac136-Ac138*, and *∆Ac148-Ac150* in histograms, respectively) at MOI 0.5 (**A**) and 5 (**B**) 72 h post-infection. Final BV titers (represented as genome copies × mL) were estimated using qPCR. The nonedited virus (*Ac-eGFP/HRPc*, “Parental” in histograms) was included as the reference. The values are the means of at least three independent assays. The error bars represent the standard error of the mean. No statistically significant differences were observed.

To characterize the infectivity of BVs and the genome replication of the edited viruses, the supernatant from Sf9 cells infected with the recombinant viruses was titrated and used to infect new cell cultures at MOI 0.5 and 5. The virus titers were estimated at 4 dpi using qPCR. No significant difference in BV production was found among the tested viruses, suggesting that none of the gene knockouts affected viral infectivity mediated by BVs. Additionally, genome multiplication in the infected cells confirmed that the set of genes affected was nonessential for AcMNPV replication (Figure 6).

### 3.3. Impact of Gene Edition by cas9 Using Dual sgRNA on Recombinant Protein Production in Insect Cells

**Figure 7:**
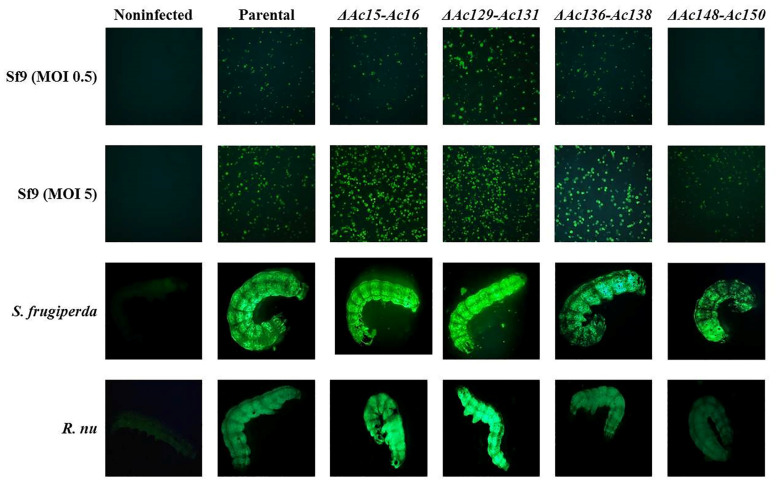
Fluorescence microscopy of Sf9 cells and larvae, expressing eGFP after infection with parental (*Ac-eGFP/HRPc*) or edited recombinant AcMNPV (*Ac-eGFP/HRPc^∆Ac15-Ac16^*, *Ac-eGFP/HRPc^∆Ac129-Ac131^*, *Ac-eGFP/HRPc^∆Ac136-Ac138^*, *Ac-eGFP/HRPc^∆Ac148-Ac150^,* mentioned as *∆Ac15-Ac16*, *∆Ac129-Ac131*, *∆Ac136-Ac138*, and *∆Ac148-Ac150*, respectively). First and second row of photographs: representative photographs (40X) of Sf9 cells infected at MOI 0.5 (First row) and MOI 5 (Second row) at 3 dpi. An image from non-infected cells is included. Third and fourth row of photographs: representative photographs (8X) of *S. frugiperda* (third row) and *R. nu* larvae (fourth row) at 4 dpi. An image from non-infected larvae is included.

**Figure 8:**
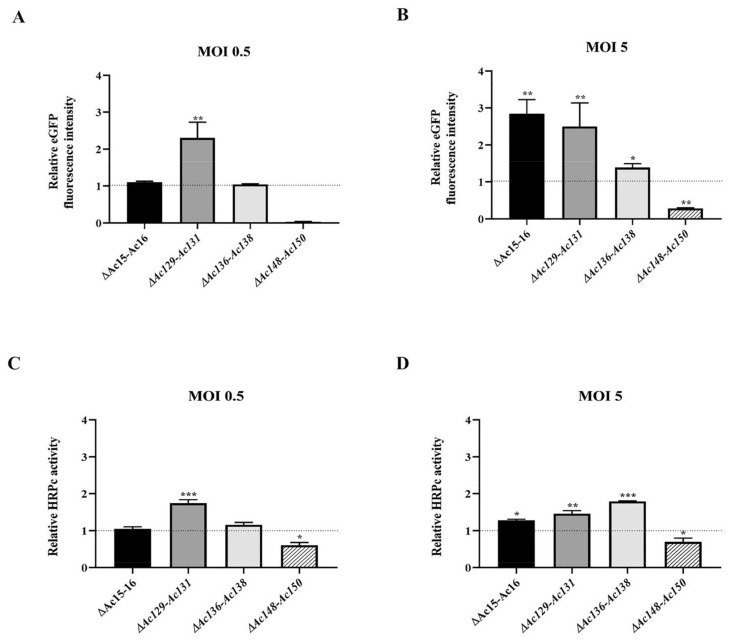
Effect of genome editing on eGFP and HRPC expression in Sf9 cells. (**A**,**B**) Analysis of eGFP expression level in Sf9 cells infected at MOI 0.5 (**A**) and 5 (**B**) with the different edited AcMNPV (*Ac-eGFP/HRPc^∆Ac15-Ac16^*, *Ac-eGFP/HRPc^∆Ac129-Ac131^*, *Ac-eGFP/HRPcC^∆Ac136-Ac138^*, and *Ac-eGFP/HRPc^∆Ac148-Ac150^*, mentioned as *∆Ac15-Ac16, ∆Ac129-Ac131*, *∆Ac136-Ac138*, and *∆Ac148-Ac150*, respectively). The eGFP production was measured as relative fluorescence intensity 72 h after infection. The results are expressed as the relative eGFP fluorescence intensity, with a value of 1 corresponding to the parental virus (nonedited, *Ac-eGFP/HRPc*). (**C**,**D**) Analysis of HRPc expression level in SF9 cells infected at MOI 0.5 (**C**) and 5 (**D**) with the different edited AcMNPV. The HRPc production was measured as relative activity (U/mL) at 96 h after infection. The results are expressed as the relative HRPc activity, with a value of 1 corresponding to the parental virus (nonedited, *Ac-eGFP/HRPc*). The values are the means of at least three independent assays. The error bars represent the standard error of the mean. Columns with an asterisk were significantly different. * *p* < 0.05; ** *p* < 0.01, *** *p* < 0.001.

Sf9 cells were infected with edited (*Ac-eGFP/HRPc^∆Ac15-Ac16^*, *Ac-eGFP/HRPc^∆Ac129-Ac131^*, *Ac-eGFP/HRPc^∆Ac136-Ac138^*, and *Ac-eGFP/HRPc^∆Ac148-Ac150^*) and nonedited (parental, *Ac-eGFP/HRPc*) recombinant baculoviruses at MOIs 0.5 and 5. The expression of the two proteins (eGFP and HRPc) was analyzed to evaluate the impact of the performed genome mutations on recombinant protein production in insect cells. At 3 dpi, cells were collected for eGFP detection, and fluorescence was monitored by microscopy (Figure 7). In addition, the infected-cell supernatants were harvested for HRPc protein detection at 4 dpi. The effect of the genome deletions on the expression of both recombinant proteins was similar in Sf9 cells infected with the different viruses. As shown in Figure 8 eGFP fluorescence (Figure 8A,B) and HRPc expression (Figure 8C,D) were significantly enhanced in Sf9 cells infected at MOI 0.5 by *Ac-eGFP/HRPc^∆Ac129-Ac131^* compared to cells infected with the parental virus *Ac-eGFP/HRPc*. However, proteins expression remained unchanged compared to the parental in cells infected with *Ac-eGFP/HRPc^∆Ac15-Ac16^* or *Ac-eGFP/HRPc^∆Ac136-Ac138^*. Moreover, cells infected with *Ac-eGFP/HRPc^∆Ac148-Ac150^* exhibited undetectable eGFP fluorescence and weak HRPc expression, suggesting considerable suppression of protein expression. In contrast, infection at MOI 5 with *Ac-eGFP/HRPc^∆Ac15-Ac16^* and *Ac-eGFP/HRPc^∆Ac129-Ac131^* resulted in a pronounced increase in eGFP expression, while infection with *Ac-eGFP/HRPc^∆Ac136-Ac138^* led to a moderate increase in expression in Sf9 cells. However, at the same MOI, only a moderate increase in HRPc expression was observed following infection with all three edited viruses (*Ac-eGFP/HRPc^∆Ac15-Ac16^*, *Ac-eGFP/HRPc^∆Ac129-Ac131^*, and *Ac-eGFP/HRPc^∆Ac136-Ac138^*). Meanwhile, infection with the *Ac-eGFP/HRPc^∆Ac148-Ac150^* mutant resulted in significantly lower eGFP and HRPc expression levels compared to infection with the parental virus. Table 2 summarizes the effect of genome editing on the expression of eGFP and HRPc in insect sf9 cells.

### 3.4. Impact of Genome Editing on Recombinant Protein Production in Insect Larvae

**Figure 9:**
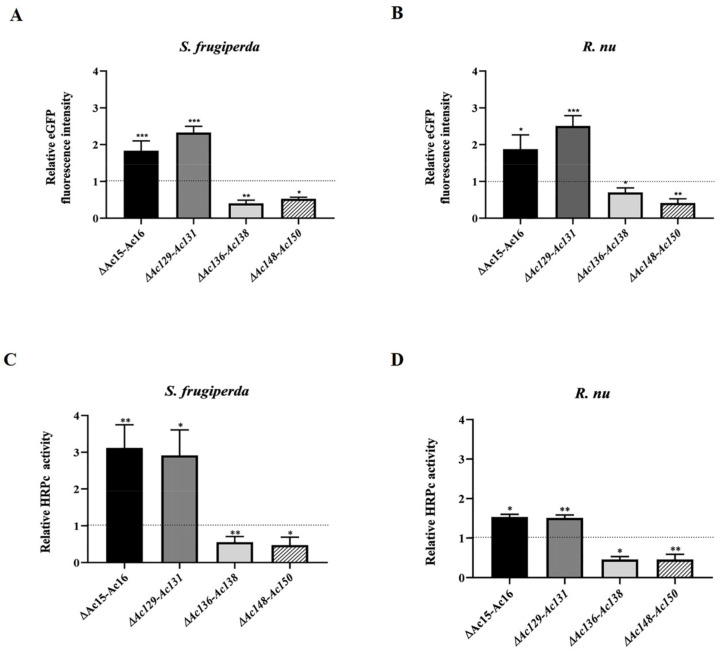
Effect of genome editing on eGFP and HRPc expression in larvae. (**A**,**B**) Analysis of eGFP expression level in *S. frugiperda* (**A**) and *R. nu* larvae (**B**) infected with the different edited AcMNPVs (*Ac-eGFP/HRPc^∆Ac15-Ac16^*, *Ac-eGFP/HRPc^∆Ac129Ac–131^*, *Ac-eGFP/HRPc^∆Ac136-Ac138^*, and *Ac-eGFP/HRPc^∆Ac148-Ac150^*, mentioned as *∆Ac15-Ac16*, *∆Ac129-Ac13*1, *∆Ac136-Ac138*, and *∆Ac148-Ac150*, respectively). The eGFP expression was measured as relative fluorescence intensity 72 h after infection. The results are expressed as the relative eGFP fluorescence intensity, with a value of 1 obtained from the parental virus (nonedited, *Ac-eGFP/HRPC*). (**C**,**D**) Analysis of HRPc expression level in *S. frugiperda* (C) and *R. nu* (D) larvae infected with the different edited AcMNPVs. The HRPc production was measured as activity (U/mL) 96 h after infection. The results are expressed as the relative HRPc activity, with a value of 1 obtained with the parental virus (nonedited). The values are the means of at least ten independent assays. The error bars represent the standard error of the mean. Columns with an asterisk were significantly different. *: *p* < 0.05; ** *p* < 0.01; *** *p* < 0.001.

The expression of eGFP and HRPc was also evaluated to determine the effects of genome editing on recombinant protein production in *S. frugiperda* and *R. nu* larvae. Larvae infected with recombinant baculoviruses were harvested at 4 dpi, and fluorescence was monitored by microscopy (Figure 7). Figure 9 shows that eGFP fluorescence (Figure 9A,B) and HRPc expression (Figure 9C,D) were significantly enhanced in *R. nu* and *S. frugiperda* larvae infected with either *Ac-eGFP/HRPc^∆Ac15-Ac16^* or *Ac-eGFP/HRPc^∆Ac129-Ac131^* compared to larvae infected with the parental *Ac-eGFP/HRPc*. Additionally, expression decreased when both insect larvae were infected with either *Ac-eGFP/HRPc^∆Ac136-Ac138^* or *Ac-eGFP/HRPc^∆Ac148-Ac150^*. Table 2 summarizes the effect of genome editing on eGFP and HRPc expression in insect larvae.

## 4. Discussion

### 4.1. Technical Considerations of Dual sgRNA-Cas9 Editing

Synthetic biology in baculovirus research enables the design and optimization of viral genomes to enhance protein expression, improve infectivity, and eliminate unwanted functions for technological applications. Through precise genome editing, synthetic baculoviruses can serve as customized vectors for various biotechnological uses, including vaccine development, gene therapy, and recombinant protein production [38]. A top-down synthetic biology approach offers the advantage of starting with a functional genome, allowing researchers to systematically remove nonessential elements while preserving key viral functions. This strategy ensures that any modifications that negatively affect viability can be reversed, optimizing the baculovirus for enhanced performance [19].

Several attempts have been made to achieve the desired minimal baculovirus genome using these strategies and evaluating their impact on expression in insect cells [11,12,39,40]. Comparative genomics studies have identified at least 38 core genes in Baculoviridae family as essential for maintaining the baculovirus life cycle in nature [10,41,42]. However, some of these genes are nonessential for BV production and are only required for ODV and OB generation [17] as *per os* infectivity factors (PIF) or polyhedrin/granulin. Additionally, proteins that manipulate or affect the host, such as egt (*Ac126*) and cathepsin (*Ac127*), are not necessary for BV production [17,21]. These findings provide the basis for developing minimized genomes by deleting nonessential genes for BV generation and propagation, enabling the incorporation of more heterologous genes of interest and enhanced protein expression levels [17]. Furthermore, transcriptomic data reveal that some of these nonessential genes exhibit high transcriptional activity [33]. Therefore, these sequences can be targeted for removal in the context of baculovirus genome optimization to enhance recombinant protein production in the BEVS.

Advances in synthetic biology tools such as CRISPR/Cas9 have accelerated the development of baculovirus-based platforms with enhanced safety, efficiency, and versatility. The CRISPR/Cas9 system was first used successfully to edit the baculovirus genome using a single sgRNA, resulting in the generation of indels [9]. Although this methodology was effective for generating knockouts, sequencing was the only reliable method for detecting mutations. Despite gene disruption, the transcriptional machinery continues to be recruited to the promoters, leading to unnecessary energy expenditure [17]. Additionally, the presence of residual nucleotide sequences can still give rise to subgenomic regulatory elements [43,44,45].

In this study, we evaluated the possibility of simultaneously editing the AcMNPV genome using Cas9 with two tandem sgRNAs, aiming to remove large DNA fragments that encode nonessential proteins for recombinant protein production in the BEVS. This strategy not only provides a rapid method for mutant detection by end-point PCR but also enables the removal of multiple tandem targets, while including promoters and regulatory regions. This facilitates the development of a minimal and optimized genome for recombinant protein production. To further enhance genome editing, we continued employing RNP delivery as previously described [9]. This method offers distinct advantages over plasmid-based methods, including rapid nuclear access of Cas9, reduced off-target effects due to its short half-life, lower cellular toxicity, and the elimination of risks associated with prolonged expression or genomic integration. Furthermore, targeting the first generation of BVs increases the likelihood of recovering mutant variants by minimizing the dilution effect during subsequent amplification steps [9,46,47]. This strategy also provides the opportunity to directly optimize the virus carrying the expression cassette or to generate an optimized, non-transposed viral genome that can be used to transform the E. coli DH10Bac^TM^ strain for the construction of new recombinant viral vectors.

A total of eleven protein genes were edited, resulting in four AcMNPV knockout viruses, *Ac-eGFP/HRPc^∆Ac15-Ac16^*, *Ac-eGFP/HRPc^∆Ac129-Ac131^*, *Ac-eGFP/HRPc^∆Ac136-Ac138^*, and *Ac-eGFP/HRPc^∆Ac148-Ac150^*. All edited fragments exhibited the expected deletion upstream of the PAM site (Figure 2, Figure 3, Figure 4 and Figure 5). A single nucleotide insertion was observed at the junction point between the two fragments in the editing target *Ac129*-Ac*131*, as well as in *Ac136*-Ac*138*. In some cases, deletion of the gene fragments resulted in pseudogenes formed by the fusion of the 5′ end of one coding sequence with the 3′ end of the adjacent gene, leading to frameshift mutations at the fusion point and the presence of premature stop codons in the predicted proteins when one of the promoter regions was retained. In other cases, a complete theoretical loss of translation occurred due to the removal of the promoter.

The mutant viruses were used to validate the editing methodology and, consequently, to evaluate their impact on foreign gene expression. For this purpose, Sf9 insect cells and *R. nu* and *S. frugiperda* larvae were infected with the recombinant viruses (edited or nonedited). These viruses carried eGFP under the late viral p10 promoter and HRPc driven by the chimeric late viral *polh-pSeL* promoter [48]. Given that expression in larvae represents a cost-effective alternative for recombinant protein production, cultured cells are still the preferred host for therapeutically relevant proteins [7]. In addition, gene deletions with distinct functions can differentially affect recombinant protein expression depending on the expression host [12,18]. This highlights the importance of evaluating their effects on protein expression in both insect cell lines and larvae.

After expression, no significant difference was found in the final BV titer achieved by parental and mutant viruses was found. This suggests that the difference in expression is not influenced by virus replication and budding (Figure 6). This is important because removing genome fragments may also eliminate sequences with unknown functions, such as miR genes or origin of replication, which could impact the viral cycle [43,44,49]. In cell culture, moderate changes in expression were observed at MOI 0.5, whereas pronounced changes became evident at a higher MOI in most of the mutants evaluated. In larvae, the effects of these same mutant viruses closely mirrored those observed in cell culture at a higher MOI, except for *Ac-eGFP/HRPc^∆136-Ac138^*, which caused increased expression in cells but decreased expression in larvae (Figure 7, Figure 8 and Figure 9). Notably, the effects of the mutations were independent of the nature of the expressed protein, whether intracellular (eGFP) or secreted (HRPc). The four gene deletions evaluated were found to have distinct effects on recombinant protein expression. Deletions of *Ac15-Ac16* and *Ac129-Ac131* consistently improved eGFP/HRPc in both cells and larvae, whereas *Ac136-Ac138* and *Ac148-Ac150* had host-specific and negative effects, respectively. The regulatory mechanisms by which these nonessential genes influence transgene expression driven by late viral promoters remain poorly understood. Therefore, further functional studies will are required to elucidate their role in modulating recombinant expression in the AcMNPV system [23].

### 4.2. Simultaneous Deletion of Ac15 and Ac16 Enhances Recombinant Protein Expression

The first gene fragment selected for removal from the parental *Ac-eGFP/HRPc* virus and used to validate the editing methodology using two gRNAs was the *Ac15* and *Ac16* ORFs. *Ac15*, which encodes Ecdysteroid UDP-glucosyltransferase (egt), is involved in arresting the molting process, and allows replication in infected larvae. Previous reports indicated that spontaneous deletions of *Ac15* during cell culture passage suggest that this gene is not essential for viral replication or survival [14]. This ORF is located adjacent to *Ac16* (ODV-E26), a gene that encodes a structural protein associated with the envelopes of both BV and ODV [50]. Although *Ac16* is involved in BV envelope formation, its deletion does not appear to affect their viability, likely because its function is compensated by the expression of other viral genes [23]. Studies have demonstrated that the inactivation of *Ac15* and Ac16 produces infective BV [14]. In this work, infections with the *Ac-eGFP/HRPc^∆Ac15-Ac16^* mutant viruses (Figure 7, Figure 8 and Figure 9) led to enhanced protein expression under conditions evaluated in Sf9 cells infected at MOI 5 and larvae as the host, except in insect cells infected at MOI 0.5, where expression levels were comparable to the infection with the parental virus. This suggests that deleting this group of genes does not substantially affect expression at a low MOI. Previously, *Ac16* was individually edited by Cas9 using a single sgRNA. This editing resulted in a mutation that introduced a premature stop codon. While knockout of the *Ac16* gene did not appear to affect GFP expression in Sf21 cells, infection of *Spodoptera exigua* larvae with the edited virus resulted in a 5-fold increase in protein production [9,14]. The strategy of simultaneously deleting more than one gene has already been reported. The deletion of *Ac15* and *Ac16* ORFs from the AcMNPV genome using the λ Red recombination benefited GFP expression in both Sf9 and High Five cells [12]. Although the results obtained in the present study were similar to those previously reported in insect cells, it was possible to demonstrate for the first time how this modification affects larval expression as well. In this study, the most pronounced enhancement in recombinant protein expression was observed when eGFP was expressed in cells infected at MOI of 5 and HRPc was expressed in *S. frugiperda* larvae, with 2.84 ± 0.38-fold and 3.11 ± 0.63-fold increases, respectively (Table 2).

### 4.3. Simultaneous Deletion of Ac129, Ac130, and Ac131 Enhances Recombinant Protein Expression

The second fragment selected in this study (*Ac129*-Ac*131*) encoded three proteins that are not essential for BV production. Specifically, *Ac129* encodes a viral capsid protein (p24) found in ODV [51]; *Ac130* (gp16) is associated with nucleocapsid formation and its transport through the nuclear membrane, as well as with the movement of BV across the nuclear envelope and into the cytoplasm [52]; and *Ac131* (Calyx; polyhedron envelope, PE; pp34) is associated with OB stability [14]. The disruption of these three genes results in viable BV production at levels similar to those of the parental virus. In addition, deletion of *Ac130* has been linked to a delayed lethal effect in larvae [52], which could be advantageous for recombinant protein production, as increased larval survival would allow for an extended production period of the target protein. In this study, eGFP expression reached approximately 2.5-fold in both insect cells and larvae. In the case of HRPc, expression increased moderately in both Sf9 cells and *R. nu* larvae. However, substantial enhancement was observed in *S. frugiperda* larvae, with expression levels reaching up to 2.91 ± 0.63-fold compared to infection with the parental virus (Figure 7, Figure 8 and Figure 9, Table 2). These results are consistent with those reported, showing that the deletion of this fragment significantly increased expression levels in Sf9 and High Five insect cells [12].

### 4.4. Divergent Expression Outcomes Following Ac136-Ac138 Fragment Deletion

Infections with the *Ac-eGFP/HRPcC^∆Ac136-Ac138^* mutant were the only cases in which protein expression exhibited the opposite behavior in insect cells and larvae (Figure 7, Figure 8 and Figure 9). In cells, this mutant moderately increased the expression of both studied proteins under most conditions evaluated, except for eGFP expression at MOI 0.5, which was comparable to the parental infection with *Ac-eGFP/HRPc*. In contrast, the expression of recombinant proteins was significantly reduced in both larval hosts. The differences in the expression levels of model proteins between Sf9 cells and larvae suggest that distinct regulatory mechanisms operate in these two biological systems. This may be due to altered activity of specific viral genes. While Sf9 cells are a homogeneous clonal cell line, larval infections occur across heterogeneous tissues. This highlights that baculoviral gene expression may vary depending on the infected cell type. Further studies are required to elucidate these findings.

This mutated virus includes the deletion of the *Ac136*, *Ac137*, and *Ac138* genes. The *Ac136* gene encodes the p26 protein, whose function remains uncharacterized. It is a secondary gene that does not influence transmission, infectivity, or production of both BV and OB. For this reason, its inactivation results in a viable virus capable of infecting insect cell culture and larvae, indicating that it is a nonessential gene for systemic infection mediated by BVs [53]. Then, *Ac137* encodes the highly expressed p10 protein, which, together with PEP, is associated with the polyhedron and plays a role in its stability. It has been observed that removal of the p10 protein does not compromise BV infectivity or replication, confirming it is a nonessential gene [54]. However, deleting it dramatically reduces virus occlusion efficiency [55]. Since p10 is highly expressed, its removal reduces competition with strong promoters that regulate recombinant protein expression [14]. Moreover, 10 proteins have been identified as essential only for oral infection and are defined as PIFs [56]. One of these proteins is codified by the *Ac138* gene, which encodes p74 (PIF-0). This protein is fundamental for midgut cell larva oral infection through ODV, but it is dispensable for virus propagation in cell culture or systemic infection in larvae [27,57]. The simultaneous expression of the three proteins ensures correct virion occlusion within the polyhedra. The commercial vector FlashBacUltra (OET) was designed with deletions of these three nonessential genes for BV production [14]. In our study, the deletion of the *Ac136*-Ac*138* fragment resulted in enhanced expression exclusively in Sf9 cells infected at MOI 5, whereas a significant reduction in expression was observed in both insect larval hosts. The partial deletion of the *Ac136*-Ac*138* fragment has been reported to positively impact the expression of recombinant proteins in the system [20]. However, it has been demonstrated that the viruses are affected when the complete coding sequence of *Ac136* and *Ac138* is deleted. In contrast, the complete deletion of *Ac137* showed no significant differences compared to the control virus [11]. In *Ac136*, the deletion might be affecting a regulatory element such as HR5, while in *Ac138*, it could be impacting the expression of the neighboring essential gene, *Ac139*, which encodes ME53. In the present study, special attention was given to neighboring genes and genetic elements. Despite this, expression was increased in cells; however, the negative impact on larvae persisted. Further studies on *Ac136* and *Ac137* are needed to determine why their deletion may be impacting expression in larvae.

### 4.5. Deletion of Ac148-Ac150 Fragment Reduces Protein Expression Despite Preserved Viral Replication

Finally, the *Ac148*-Ac*150* fragment was removed from the parental virus. The *Ac148* (encoding PIF-5/ODV-E56) and *Ac150* genes encode proteins associated with the envelopes of both ODV and BV. However, their absence dramatically reduces ODV oral infectivity in larvae, while maintaining comparable infectivity to the wild type when BVs are used to infect insect cells in culture or larvae via intrahemocoelic injection [58]. PIF-5 is specifically involved in oral infectivity. The third gene deleted in this fragment group was *Ac149*. Although little is known about this gene in AcMNPV, studies in related baculoviruses, such as in the nucleopolyhedrovirus of Bombyx mori (*BmNPV*), suggest that it may not be essential [14]. In our study, infections with the *Ac-eGFP/HRPc^∆Ac148-Ac150^* mutant led to significantly decreased expression in all studied cases (Figure 7, Figure 8 and Figure 9). Individual gene deletions had no effect on protein expression [11]. However, it was demonstrated that simultaneously removing all three genes negatively impacts protein expression in insect cells, although these mutant viruses exhibit a replicative behavior similar to the nonedited parental virus [12]. In the present study, this negative effect was also observed in insect larvae. This finding reinforces the importance of considering synergistic gene functions and regulatory context in genome minimization efforts.

## 5. Conclusions

This study successfully applied CRISPR/Cas9 genome editing using two tandem sgRNAs to systematically remove nonessential gene fragments (for biotechnological applications based on the BV morphotype) from the AcMNPV genome. This enabled the development of minimized viral vectors optimized for recombinant protein production in BEVS. The results highlight the power of rational genome engineering to enhance vector performance, as well as the importance of evaluating each genomic modification across different hosts and conditions to ensure consistent and predictable expression outcomes.

This approach can be used to enhance the productivity of BEVS by selectively modifying nonessential viral genes that modulate recombinant expression for budded virion generation. Given the scalability of insect cell cultures and larvae as live bioreactors, and the regulatory acceptance of baculovirus platforms for vaccine and biopharmaceutical production, the ability to fine-tune the viral genome introduces a powerful tool for process optimization. Furthermore, the adaptability of the CRISPR/Cas9-mediated editing workflow enables rapid generation of customized viral backbones, with the potential to improve yield, stability, and product quality across a wide range of recombinant proteins. This strategy provides a robust platform for the optimized expression of biotechnologically relevant proteins in both research and industrial settings.

In future research, we plan to sequentially perform genomic deletions that have positively impacted foreign protein expression (without compromising BV replication or production). This will allow us to evaluate potential synergistic effects that could lead to even greater benefits. In addition, we plan to test the observed impacts on proteins of biotechnological interest.

## Figures and Tables

**Table 1:** Summary of sgRNA characteristics.

Target ORF	sgRNA sequence 5′–3′	Localization	Strand	GC (%)	Efficiency
*Ac-15* (egt)	GTTTGGTCACTTGTACGATC	444	+	45	0.52
*Ac-16* (ODV-26)	GTTCACAGAACCGACCGGCA	71	-	60	0.61
*Ac-129* (p24)	GTCATCATTACCAATTCGGA	73	+	40	0.64
*Ac-131* (pp34)	AAATGTGCTCAACAACTGGT	239	-	40	0.66
*Ac-136* (p26)	AATAGAGCAAGTCGACAATG	51	+	50	0.72
*Ac-138* (p74)	AACTGGCTTTCAGCAAGCGC	191	-	55	0.62
*Ac148*(ODV-E56)	CTTTTAACAAGCACTCCCGC	79	-	50	0.62
*Ac-150*	GACGATGACGAATCAGACGA	91	+	50	0.68

Localization: From the sequence next to the protospacer adjacent motif (PAM) site to the start ATG codon; + indicates the coding DNA strand; - indicates complementary DNA strand. Efficiency score (normalized between 0 and 1) estimates the cutting efficiency of sgRNAs using sequence-based predictive models. All the parameters were provided by the CHOPCHOP bioinformatics tool.

**Table 2:** Summary of the effects of genome editing on eGFP and HRPc expression.

Edited Virus	Host	Impact on eGFP Level Expression (Fold Change) Relative to Parental Virus	Impact on HRPc Level Expression (Fold Change) Relative to Parental Virus
Ac-eGFP/HRPc^∆Ac15-Ac16^	Sf9 at MOI 0.5	NS	NS
	Sf9 at MOI 5	↑2.84 ± 0.38	↑1.27 ± 0.02
	S. frugiperda	↑1.84 ± 0.26	↑3.11 ± 0.63
	R. nu	↑1.87 ± 0.30	↑1.54 ± 0.06
Ac-eGFP/HRPc^∆Ac129-Ac131^	Sf9 at MOI 0.5	↑2.30 ± 0.41	↑1.74 ± 0.09
	Sf9 at MOI 5	↑2.5 ± 0.62	↑1.46 ± 0.07
	S. frugiperda	↑2.3 ± 0.17	↑2.91 ± 0.63
	R. nu	↑2.5 ± 0.28	↑1.51 ± 0.07
Ac-eGFP/HRPc^∆Ac136-Ac138^	Sf9 at MOI 0.5	NS	NS
	Sf9 at MOI 5	↑1.40 ± 0.10	↑1.79 ± 0.01
	S. frugiperda	↓0.41 ± 0.08	↓0.55 ± 0.15
	R. nu	↓0.70 ± 0.12	↓0.46 ± 0.07
Ac-eGFP/HRPc^∆Ac148-Ac150^	Sf9 at MOI 0.5	ND	↓0.60 ± 0.07
	Sf9 at MOI 5	↓0.29 ± 0.02	↓0.70 ± 0.10
	S. frugiperda	↓0.52 ± 0.04	↓0.47 ± 0.15
	R. nu	↓0.41 ± 0.11	↓0.45 ± 0.12

Values > 1 indicate upregulation (↑); values < 1 indicate downregulation (↓). NS: non-significant difference relative to *Ac-eGFP/HRPc* parental virus (*p* < 0.5) ND: not detected. The values are the means of at least three independent assays. The error represent the standard error of the mean.

## Data Availability

The original contributions presented in this study are included in the article. Further inquiries can be directed to the corresponding authors.

## Supplementary Materials

The following supporting information can be downloaded from: https://www.mdpi.com/article/10.3390/v17081041/s1, Figure S1: In vitro evaluation of sgRNA Ac15, Ac16, Ac148 and Ac150. Figure S2: In vitro evaluation of sgRNA Ac129 and Ac131. Figure S3: In vitro evaluation of sgRNA Ac136 and Ac138. Table S1: Summary of PCR primers employed in the study. Table S2: Predicted promoters by bioinformatics analysis.

## Abbreviations

The following abbreviations are used in this manuscript:

AcMNPV	Autographa californica Nuclear Polyhedrosis Virus
BEVS	Baculovirus Expression Vector System
BV	Budded viruses
DPI	Days post-infection
DI_50_	Infectious dose 50
DSB	DNA double-strand break
eGFP	Enhanced green fluorescent protein
FBS	Fetal bovine serum
FU	Fluorescent unit
GP64	Glycoprotein 64 leader peptide
HRPc	Horseradish peroxidase isoenzyme C
HR5	Homologous region 5
ND	Not detected
NS	Non-significant
ODV	Occlusion-derived viruses
OB	Budded viruses
ORF	Open reading frames
PAM	Protospacer adjacent motif
PIF	*Per os* infectivity factors
PCR	Polymerase chain reaction
*R. nu*	*Rachiplusia nu*
*S. frugiperda*	*Spodoptera frugiperda*
PFU	Plaque-forming units
qPCR	Quantitative real-time PCR
RNP	Ribonucleoprotein
sgRNA	Single-guide RNA

## References

[1] Targovnik A.M., Simonin J.A., Mc Callum G.J., Smith I., Cuccovia Warlet F.U., Nugnes M.V., Miranda M.V., Belaich M.N. (2021). Solutions against Emerging Infectious and Noninfectious Human Diseases through the Application of Baculovirus Technologies. Appl. Microbiol. Biotechnol..

[2] Ikonomou L., Schneider Y.J., Agathos S.N. (2003). Insect Cell Culture for Industrial Production of Recombinant Proteins. Appl. Microbiol. Biotechnol..

[3] Hong M., Li T., Xue W., Zhang S., Cui L., Wang H., Zhang Y., Zhou L., Gu Y., Xia N. (2022). Genetic Engineering of Baculovirus-Insect Cell System to Improve Protein Production. Front. Bioeng. Biotechnol..

[4] Felberbaum R.S. (2015). The Baculovirus Expression Vector System: A Commercial Manufacturing Platform for Viral Vaccines and Gene Therapy Vectors. Biotechnol. J..

[5] Airenne K.J., Hu Y.C., Kost T.A., Smith R.H., Kotin R.M., Ono C., Matsuura Y., Wang S., Ylä-Herttuala S. (2013). Baculovirus: An Insect-Derived Vector for Diverse Gene Transfer Applications. Mol. Ther..

[6] Sokolenko S., George S., Wagner A., Tuladhar A., Andrich J.M.S., Aucoin M.G. (2012). Co-Expression vs. Co-Infection Using Baculovirus Expression Vectors in Insect Cell Culture: Benefits and Drawbacks. Biotechnol. Adv..

[7] Martínez-Solís M., Herrero S., Targovnik A.M. (2019). Engineering of the Baculovirus Expression System for Optimized Protein Production. Appl. Microbiol. Biotechnol..

[8] Chen Y.-R., Zhong S., Fei Z., Hashimoto Y., Xiang J.Z., Zhang S., Blissard G.W. (2013). The Transcriptome of the Baculovirus Autographa Californica Multiple Nucleopolyhedrovirus in Trichoplusia Ni Cells. J. Virol..

[9] Pazmiño-Ibarra V., Mengual-Martí A., Targovnik A.M., Herrero S. (2019). Improvement of Baculovirus as Protein Expression Vector and as Biopesticide by CRISPR/Cas9 Editing. Biotechnol. Bioeng..

[10] Cerrudo C.S., Motta L.F., Uriel F., Warlet C., Lassalle F.M., Simonin J.A., Nicol M. (2023). Protein-Gene Orthology in Baculoviridae: An Exhaustive Analysis to Redefine the Ancestrally Common Coding Sequences. Viruses.

[11] Yu Y., Zhang T., Lu D., Wang J., Xu Z., Zhang Y., Liu Q. (2023). Genome-Wide Nonessential Gene Identification of Autographa Californica Multiple Nucleopolyhedrovirus. Gene.

[12] Zhang X., He A., Zong Y., Tian H., Zhang Z. (2023). Improvement of Protein Production in Baculovirus Expression Vector System by Removing a Total of 10 Kb of Nonessential Fragments from Autographa Californica Multiple Nucleopolyhedrovirus Genome. Front. Microbiol..

[13] Shrestha A., Bao K., Chen Y.-R., Chen W., Wang P., Fei Z., Blissard G.W. (2018). Global Analysis of Baculovirus Autographa Californica Multiple Nucleopolyhedrovirus Gene Expression in the Midgut of the Lepidopteran Host Trichoplusia Ni. J. Virol..

[14] Rohrmann G. (2019). Baculovirus Molecular Biology.

[15] Targovnik A.M., Arregui M.B., Bracco L.F., Urtasun N., Baieli M.F., Segura M.M., Simonella M.A., Fogar M., Wolman F.J., Cascone O. (2016). Insect Larvae: A New Platform to Produce Commercial Recombinant Proteins. Curr. Pharm. Biotechnol..

[16] Kato T., Kajikawa M., Maenaka K., Park E.Y. (2010). Silkworm Expression System as a Platform Technology in Life Science. Appl. Microbiol. Biotechnol..

[17] Wang M., Hu Z. (2020). Advances in the Molecular Biology of Baculoviruses. Curr. Issues Mol. Biol..

[18] Chen T., Duan X., Hu H., Shang Y., Hu Y., Deng F., Wang H. (2021). Systematic Analysis of 42 Autographa Californica Multiple Nucleopolyhedrovirus Genes Identifies An Additional Six Genes Involved in the Production of Infectious Budded Virus. Virol. Sin..

[19] Wang L., Maranas C.D. (2018). MinGenome: An in Silico Top-Down Approach for the Synthesis of Minimized Genomes. ACS Synth. Biol..

[20] Hitchman R.B., Possee R.D., Crombie A.T., Chambers A., Ho K., Siaterli E., Lissina O., Sternard H., Novy R., Loomis K. (2010). Genetic Modification of a Baculovirus Vector for Increased Expression in Insect Cells. Cell Biol. Toxicol..

[21] Kaba S.A., Salcedo A.M., Wafula P.O., Vlak J.M., Van Oers M.M. (2004). Development of a Chitinase and V-Cathepsin Negative Bacmid for Improved Integrity of Secreted Recombinant Proteins. J. Virol. Methods.

[22] Lin C., Li H., Hao M., Xiong D., Luo Y., Huang C., Yuan Q., Zhang J., Xia N. (2016). Increasing the Efficiency of CRISPR/Cas9-Mediated Precise Genome Editing of HSV-1 Virus in Human Cells. Sci. Rep..

[23] Cao Z., Liu X., Li J., Zheng Y., Yin J., Wang H., Zhang X., Chen H. (2025). Construction of a Shortened Autographa Californica Multiple Nucleopolyhedrovirus Genome as Protein Expression Vector. Arch. Virol..

[24] Pelosse M., Crocker H., Gorda B., Lemaire P., Rauch J., Berger I. (2017). MultiBac: From Protein Complex Structures to Synthetic Viral Nanosystems. BMC Biol..

[25] Datsenko K.A., Wanner B.L. (2000). One-Step Inactivation of Chromosomal Genes in Escherichia Coli K-12 Using PCR Products. Proc. Natl. Acad. Sci. USA.

[26] Li K.C., Chang Y.H., Hsu M.N., Lo S.C., Li W.H., Hu Y.C. (2017). Baculovirus-Mediated MiR-214 Knockdown Shifts Osteoporotic ASCs Differentiation and Improves Osteoporotic Bone Defects Repair. Sci. Rep..

[27] Nugnes M.V., Targovnik A.M., Mengual-Martí A., Miranda M.V., Cerrudo C.S., Herrero S., Belaich M.N. (2021). The Membrane-Anchoring Region of the Acmnpv P74 Protein Is Expendable or Interchangeable with Homologs from Other Species. Viruses.

[28] Hou S., Chen X., Wang H., Tao M., Hu Z. (2002). Efficient Method to Generate Homologous Recombinant Baculovirus Genomes in E. Coli. Biotechniques.

[29] Targovnik A.M., Ferrari A., Mc Callum G.J., Arregui M.B., Smith I., Bracco L.F., Alfonso V., López M.G., Martínez-Solís M., Herrero S. (2019). Highly Efficient Production of Rabies Virus Glycoprotein G Ectodomain in Sf9 Insect Cells. 3 Biotech.

[30] Poodts J., Smith I., Birenbaum J.M., Rodriguez M.S., Montero L., Wolman F.J., Marfía J.I., Valdez S.N., Alonso L.G., Targovnik A.M. (2022). Improved Expression of SARS-CoV-2 Spike RBD Using the Insect Cell-Baculovirus System. Viruses.

[31] Vaughn J.L., Goodwin R.H., Tompkins G.J., McCawley P. (1977). The Establishment of Two Cell Lines from the Insect Spodoptera Frugiperda (Lepidoptera; Noctuidae). In Vitro.

[32] Graentzdoerffer A. (2003). Titration of Non-Occluded Baculovirus Using a Cell Viability Assay. Biotechniques.

[33] Martínez-Solís M., Jakubowska A.K., Herrero S. (2017). Expression of the Lef5 Gene from Spodoptera Exigua Multiple Nucleopolyhedrovirus Contributes to the Baculovirus Stability in Cell Culture. Appl. Microbiol. Biotechnol..

[34] O’Reilly D.R., Miller L.K., Luckow V.A. (1994). Baculovirus Expression Vector: A Laboratory Manual.

[35] Giménez C.S., Castillo M.G., Simonin J.A., Núñez Pedrozo C.N., Pascuali N., Bauzá M.d.R., Locatelli P., López A.E., Belaich M.N., Mendiz A.O. (2020). Effect of Intramuscular Baculovirus Encoding Mutant Hypoxia-Inducible Factor 1-Alpha on Neovasculogenesis and Ischemic Muscle Protection in Rabbits with Peripheral Arterial Disease. Cytotherapy.

[36] Tjissen P., Burdon R.H., van Knippenberg P.H. (1985). Practice and Theory of Enzyme Immunoassays.

[37] Do P.T., Nguyen C.X., Bui H.T., Tran L.T.N., Stacey G., Gillman J.D., Zhang Z.J., Stacey M.G. (2019). Demonstration of Highly Efficient Dual GRNA CRISPR/Cas9 Editing of the Homeologous GmFAD2-1A and GmFAD2-1B Genes to Yield a High Oleic, Low Linoleic and α-Linolenic Acid Phenotype in Soybean. BMC Plant Biol..

[38] Chang C.-W., Wang L.-S., Pham N.N., Shen C.-C., Hsu M.-N., Nguyen N.T.K., Yen C.-Y., Lin M.-W., Hwu J.-R., Chang Y.-H. (2022). Synthetic Biology Approach to Developing All-in-One Baculovirus Vector Using Mammalian Introns and MiRNA Binding Sites. J. Taiwan Inst. Chem. Eng..

[39] Guo Y., Hu H., Xiao H., Deng F., Li J., Wang M., Hu Z. (2022). Successful Rescue of Synthetic AcMNPV with a ~17 Kb Deletion in the C1 Region of the Genome. Viruses.

[40] Shang Y., Wang M., Xiao G., Wang X., Hou D., Pan K., Liu S., Li J., Wang J., Arif B.M. (2017). Construction and Rescue of a Functional Synthetic Baculovirus. ACS Synth. Biol..

[41] Garavaglia M.J., Miele S.A.B., Iserte J.A., Belaich M.N., Ghiringhelli P.D. (2012). The Ac53, Ac78, Ac101, and Ac103 Genes Are Newly Discovered Core Genes in the Family Baculoviridae. J. Virol..

[42] Javed M.A., Biswas S., Willis L.G., Harris S., Pritchard C., van Oers M.M., Donly B.C., Erlandson M.A., Hegedus D.D., Theilmann D.A. (2017). Autographa Californica Multiple Nucleopolyhedrovirus AC83 Is a Per Os Infectivity Factor (PIF) Protein Required for Occlusion-Derived Virus (ODV) and Budded Virus Nucleocapsid Assembly as Well as Assembly of the PIF Complex in ODV Envelopes. J. Virol..

[43] Jiao Y., Wang J., Deng R., Yu X., Wang X. (2019). AcMNPV-MiR-3 Is a MiRNA Encoded by Autographa Californica Nucleopolyhedrovirus and Regulates the Viral Infection by Targeting Ac101. Virus Res..

[44] Miele S.A.B., Garavaglia M.J., Belaich M.N., Ghiringhelli P.D. (2011). Baculovirus: Molecular Insights on Their Diversity and Conservation. Int. J. Evol. Biol..

[45] Oliveira H.d.P., dos Santos E.R., Harrison R.L., Ribeiro B.M., Ardisson-Araújo D.M.P. (2022). Identification and Analysis of Putative TRNA Genes in Baculovirus Genomes. Virus Res..

[46] Kim S., Kim D., Cho S.W., Kim J., Kim J.S. (2014). Highly Efficient RNA-Guided Genome Editing in Human Cells via Delivery of Purified Cas9 Ribonucleoproteins. Genome Res..

[47] Liang X., Potter J., Kumar S., Zou Y., Quintanilla R., Sridharan M., Carte J., Chen W., Roark N., Ranganathan S. (2015). Rapid and Highly Efficient Mammalian Cell Engineering via Cas9 Protein Transfection. J. Biotechnol..

[48] Martínez-Solís M., Gómez-Sebastián S., Escribano J.M., Jakubowska A.K., Herrero S. (2016). A Novel Baculovirus-Derived Promoter with High Activity in the Baculovirus Expression System. PeerJ.

[49] Huang H., Wang M., Deng F., Wang H., Hu Z. (2012). ORF85 of HearNPV Encodes the per Os Infectivity Factor 4 (PIF4) and Is Essential for the Formation of the PIF Complex. Virology.

[50] Burks J.K., Summers M.D., Braunagel S.C. (2007). BV/ODV-E26: A Palmitoylated, Multifunctional Structural Protein of Autographa Californica Nucleopolyhedrovirus. Virology.

[51] Kokusho R., Katsuma S. (2021). Loss of P24 from the Bombyx Mori Nucleopolyhedrovirus Genome Results in the Formation of Cuboidal Occlusion Bodies. Virology.

[52] Yang M., Huang C., Qian D., Li L. (2014). Functional Characterization of Autographa Californica Multiple Nucleopolyhedrovirus Gp16 (Ac130). Virology.

[53] Simón O., Williams T., Caballero P., Possee R.D. (2008). Effects of Acp26 on In Vitro and In Vivo Productivity, Pathogenesis and Virulence of Autographa Californica Multiple Nucleopolyhedrovirus. Virus Res..

[54] Lee S.Y., Poloumienko A., Belfry S., Qu X., Chen W., MacAfee N., Morin B., Lucarotti C., Krause M. (1996). A Common Pathway for P10 and Calyx Proteins in Progressive Stages of Polyhedron Envelope Assembly in AcMNPV-Infected Spodoptera Frugiperda Larvae. Arch. Virol..

[55] Wang L., Salem T.Z., Campbell D.J., Turney C.M., Kumar C.M.S., Cheng X.W. (2009). Characterization of a Virion Occlusion-Defective Autographa Californica Multiple Nucleopolyhedrovirus Mutant Lacking the P26, P10 and P74 Genes. J. Gen. Virol..

[56] Li Z., Zhang N., Zhang T., Wang Z., Li J., Wang M., Hu Z., Wang X. (2024). AcMNPV P74 Is Cleaved at R325 and R334 by Proteinases of Both OB and BBMV to Expose a Potential Fusion Peptide for Oral Infection. J. Virol..

[57] Faulkner P., Kuzio J., Williams G.V., Wilson J.A. (1997). Analysis of P74, a PDV Envelope Protein of Autographa Californica Nucleopolyhedrovirus Required for Occlusion Body Infectivity in Vivo. J. Gen. Virol..

[58] Lapointe R., Popham H.J.R., Straschil U., Goulding D., O’Reilly D.R., Olszewski J.A. (2004). Characterization of Two Autographa Californica Nucleopolyhedrovirus Proteins, Ac145 and Ac150, Which Affect Oral Infectivity in a Host-Dependent Manner. J. Virol..

