# Supplementary material for: CRISPR/Cas9-Driven Engineering of AcMNPV Using Dual gRNA for Optimized Recombinant Protein Production
Source: Viruses. 2025 Jul 25;17(8):1041. doi: 10.3390/v17081041 (PMC12390737; doi:10.3390/v17081041)
Supplement: Supplementary file 1 [file viruses-17-01041-s001.zip › viruses-3749061-supplementary.pdf]

Supplementary Material

A

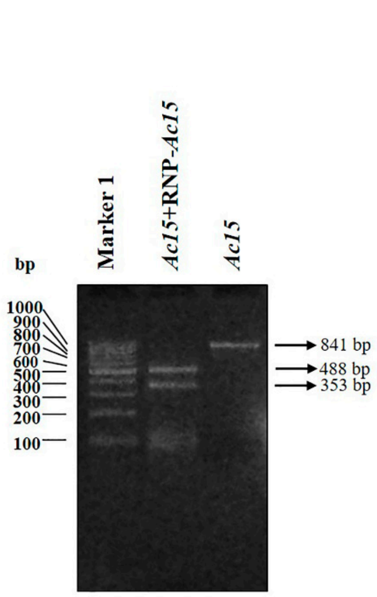

B

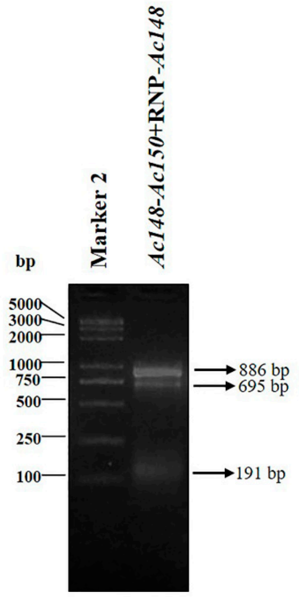

C

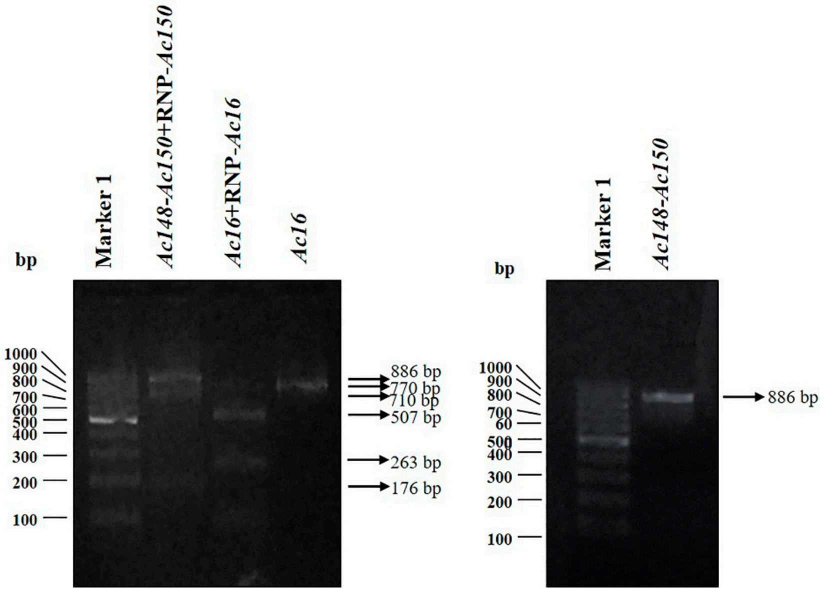

Figure S1. *In vitro* evaluation of sgRNA *Ac15*, *Ac16*, *Ac148* and *Ac150*. **A.** *In vitro* evaluation of sgRNA *Ac15*. The lane labeled "*Ac15*" corresponds to the non-digested *Ac15* PCR fragment. The lane labeled "*Ac15*+RNP-*Ac15*" corresponds to the *Ac15* fragment digested with RNP-*Ac15* (sgRNA *Ac15* +Cas9). For *Ac15* PCR amplification, specific primers were used (Fw-*Ac15* and Rev-*Ac15*). The digestion of the *Ac15* fragment (841

bp) resulted in two fragments: 488 bp and 353 bp. *B. In vitro* evaluation of sgRNA *Ac148*. The line labeled "*Ac148-Ac150*+RNP-*Ac148*" corresponds to the *Ac148-Ac150* fragment digested with RNP-*Ac148* (sgRNA *Ac148* +Cas9). The digestion of the *Ac148-Ac150* fragment (886 bp) resulted in two fragments: 695 bp and 191 bp. **C.** *In vitro* evaluation of sgRNA *Ac150* and *Ac16*. The lane labeled "*Ac-148-Ac150*" corresponds to the non-digested *Ac148-150* PCR fragment. The lane labeled "*Ac148-Ac150*+RNP-*Ac150*" corresponds to the *Ac148-Ac150* fragment digested with RNP-*Ac150* (sgRNA *Ac150* +Cas9). The lane labeled "*Ac16*" corresponds to the non-digested *Ac16* PCR fragment. The lane labeled "*Ac16*+RNP-*Ac16*" corresponds to the *Ac16* fragment digested with RNP-*Ac16* (sgRNA *Ac16* +Cas9). For *Ac148-Ac150* PCR amplification, specific primers were used (Fw-*Ac148* and Rev-*Ac150*). The digestion of the *Ac148-Ac150* fragment (886 bp) resulted in two fragments: 710 bp and 176 bp. For *Ac16* PCR amplification, specific primers were used (Fw-*Ac16* and Rev-*Ac16*). The digestion of the *Ac16* fragment (770 bp) resulted in two fragments: 507 bp and 263 bp. Marker 1: 1000 bp (Bio Basic Inc., Ontario, Canada). Marker 2: Trans 2k plus (Transgen Biotech).

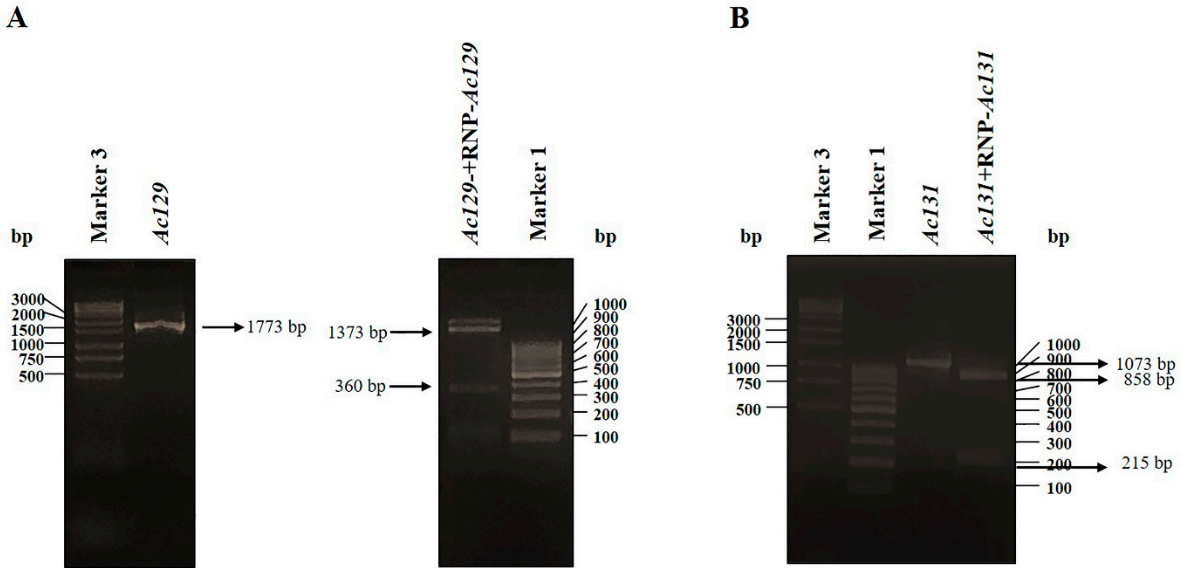

Figure S2. *In vitro* evaluation of sgRNA *Ac129* and *Ac131*. **A.** *In vitro* evaluation of sgRNA *Ac129*. The line labeled "*Ac129*" corresponds to the non-digested *Ac129* PCR fragment. The line labeled "*Ac129*+RNP-*Ac129*" corresponds to the *Ac129* fragment digested with RNP-*Ac129 (sgRNA-Ac129* +Cas9). For *Ac129* PCR amplification, specific primers were used (Fw-*Ac129* and Rev-*Ac130*). The digestion of the *Ac129-Ac131* fragment (1733 bp) resulted in two fragments: 1373 bp and 360 bp. **B.** *In vitro* evaluation of sgRNA *Ac131*. The line labeled "*Ac131*" corresponds to the non-digested *Ac131* PCR fragment. The line labeled "*Ac131*+RNP-*Ac131*" corresponds to the *Ac131* fragment digested with RNP-*Ac131* (sgRNA *Ac131*+Cas9). For *Ac131* PCR amplification, specific primers were used (Fw-*Ac131* and Rev-*Ac131*). The digestion of the *Ac131* fragment (1073 bp) resulted in two fragments: 215 bp and 858 bp. Marker 1:1000 bp (Biobasic); Marker 3: Ladder 1 kpb *plus* (Productos Biologicos, Buenos Aires, Argentina).

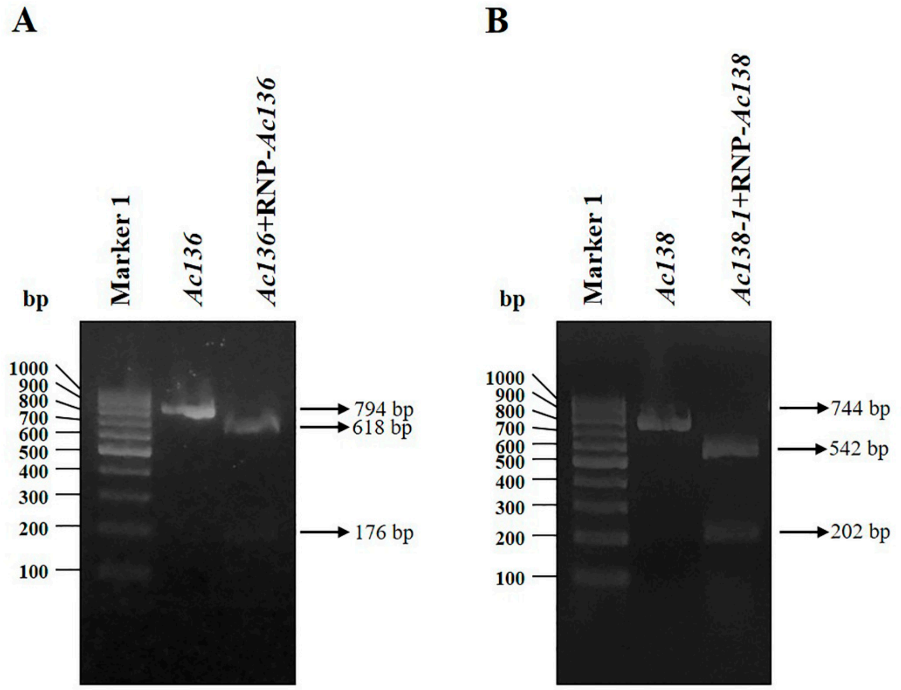

Figure S3. In vitro evaluation of sgRNA *Ac136* and *Ac138*. A. In vitro evaluation of sgRNA *Ac136*. The Lane labeled "*Ac136*" corresponds to the non-digested *Ac136* PCR fragment. The lane labeled "*Ac136*+RNP-*Ac136*" corresponds to the *Ac136* fragment digested with RNP-*Ac136* (sgRNA-*Ac136* +Cas9). For *Ac136* PCR amplification, specific primers were used (Fw-*Ac136* and Rev-*Ac136*). The digestion of the *Ac136* fragment (794 bp) resulted in two fragments: 618 bp and 176 bp. **B.** In vitro evaluation of sgRNA *Ac138*. The laine labeled "*Ac138*" corresponds to the non-digested *Ac138* PCR fragment. The lane labeled "*Ac138*+RNP-*Ac138*" corresponds to the *Ac138* fragment digested with RNP-*Ac138* (sgRNA-*Ac138* +Cas9). For *Ac138* PCR amplification, specific primers were used (Fw-*Ac138* and Rev-*Ac138*). The digestion of the *Ac138* PCR fragment (744 bp) resulted in two fragments: 542 bp and 202 bp. Marker 1: 1000 bp (Biobasic).

Table S1. Summary of PCR primers employed in the study

Name	Sequence (5-3´)
Fw-HRPcEcoR1	CGGAATTCATGCTACTAGTAAATCAGTCAC
Rv-HRPcEcoR1	CGGAATTCTCATCGCCGACGTCGTCTC
Fw-*Ac15*	CCAGTACAGTTATTCGGTTTGAAG
Rv-*Ac15*	GCTCTTTACAAGATGGATTCCTCC
Fw-*Ac16*	CGTTTCCAGCGATCAACTAC
Rv-*Ac16*	TCTGTGCGTTGTCTTCTTCTGT
Fw-*Ac129*	GTCTTCATTTGCGCGTTGCA
Fw-*Ac131*	GATTCAGGAGAGTCTCAACG
Rv-*Ac131*	GAATATTTGTCGACGCCCTC
Fw-*Ac136*	GCACATGGCTCATAACTAAAC
Rev-*Ac136*	CCGGCATCCTCAAATGCATA
Fw-*Ac138*	AGTATGCTGGAAGGCGCTTT
Rv-*Ac138*	CGGTTTTAACAGCCGTCGAT
Fw-*Ac148*	GGTCTGAAATGCCCTGAAATAC
Rv-*Ac150*	AGTTTTGGTTAGCGGTACATCC
Fw-ie1	ACCATCGCCCAGTTCTGCTTATC
Rv-ie1	GCTTCCGTTTAGTTCCAGTTGCC

Fw: Forward. Rv: Reverse.

Table S2: Predicted promoters by bioinformatics analysis

ORF	Predicted Promoter	Position from ATG start codon	Score
Ac14	ACACGGCCAATATATTGGCCGCATTTACAGCAGTAAGCGTAGACAGCAGT	-185	0.95
Ac15	AATTTTTGGCTATAAAAAGGTCACCCTTTAAAATTTGTTACATAATCAAA	-81	0.95
Ac16	ACTTCCAGCCTTTATAAACGCTCACCAACCAAAGCAGGTCATTATTGTGC	-77	0.99
Ac129	CAAAAGCACATATAAAACAATAGCGCTTACCATCTTGCTTGTGTGTTCCT	-215	0.93
Ac130	ND	-----	-----
Ac131	AATTGATTCTCTTAAAACGGACACGTTTATGATGTTGAGCAACTTGCAAA	-234	0.94
	GACGCAGTTGTAAAAAATGGCAAAAAAATATCCAATCTCGACGAAAAAAT	-163	0.92
Ac137	CGTCCGTGTTGAAAAATGGCGACCCAACATGTCCGTCTACGGGACTGTGC	-313	0.99
Ac138	AACTGTATTATAAAAATCCCGTGTTATATTGTACCAAATGCGGGTTTACC	-326	0.9
Ac148	TGAAACGGTATATAAATTTAGCGATCATACAATGGAGAGATATCATCCGT	-311	0.96
Ac149	ND	-----	-----
Ac150	TATTATCAGTAATAAAACTGGCCTGATTAGGATACAATTTATTGACTGCG	-390	0.96

Bioinformatics analysis was performed using Fruitfly platform (https://www.fruitfly.org/seq_tools/promoter.html; last accessed on 15 June 2025). Only the most probable promoters are reported. ND: no data (promoter not predicted)
